# Development of durable sulfur nanoparticle-based antifungal coatings for cotton fabrics

**DOI:** 10.1039/d6ra05516h

**Published:** 2026-09-21

**Authors:** Nirasha Jayawardhana, Kasindu Pramod, Danushika C. Manatunga, Nuwan De Silva

**Affiliations:** a Department of Biosystems Technology, Faculty of Technology, University of Sri Jayewardenepura Homagama 10206 Sri Lanka danushi@sjp.ac.lk; b Sri Lanka Institute of Nanotechnology (Pvt) Ltd (SLINTEC) Homagama 10206 Sri Lanka

## Abstract

Cotton fabrics are widely used in the textile industry due to their comfort and versatility; however, their high moisture absorption makes them highly susceptible to fungal growth, leading to material degradation and hygiene concerns. In this study, a durable antifungal coating for 100% cotton woven fabric was developed using sulfur nanoparticles (SNPs) as a low-toxicity and environmentally friendly alternative to conventional antifungal agents. SNPs were synthesized following chemical precipitation to evaluate their suitability for textile functionalization. The treated fabrics were prepared using the exhaustion method to ensure effective nanoparticle uptake and distribution within the fibre structure. Antifungal performance was assessed against *Aspergillus niger* using the AATCC TM 30 standard test method. The SNP-treated fabrics exhibited strong antifungal activity, with significant inhibition of fungal growth compared to untreated samples. The durability of the coating was evaluated after 5 and 10 laundering cycles, and the treated fabrics retained substantial antifungal activity even after repeated washing. In particular, the persistence of inhibition zones after 10 cycles indicates effective adhesion of SNPs to the cotton fibres and highlights the potential for long-term application. The developed SNP-based treatment provides an effective, durable and metal-free antifungal solution for cotton textiles. The findings demonstrate the potential of SNPs as a sustainable alternative to conventional antifungal finishes, with promising implications for hygienic and high-performance textile applications in health care and personal protective equipment (PPE).

## Abstract 2

Durable sulfur nanoparticle-coated cotton fabrics demonstrating effective antifungal activity against *Aspergillus niger* after repeated washing cycles.

## Graphical abstract

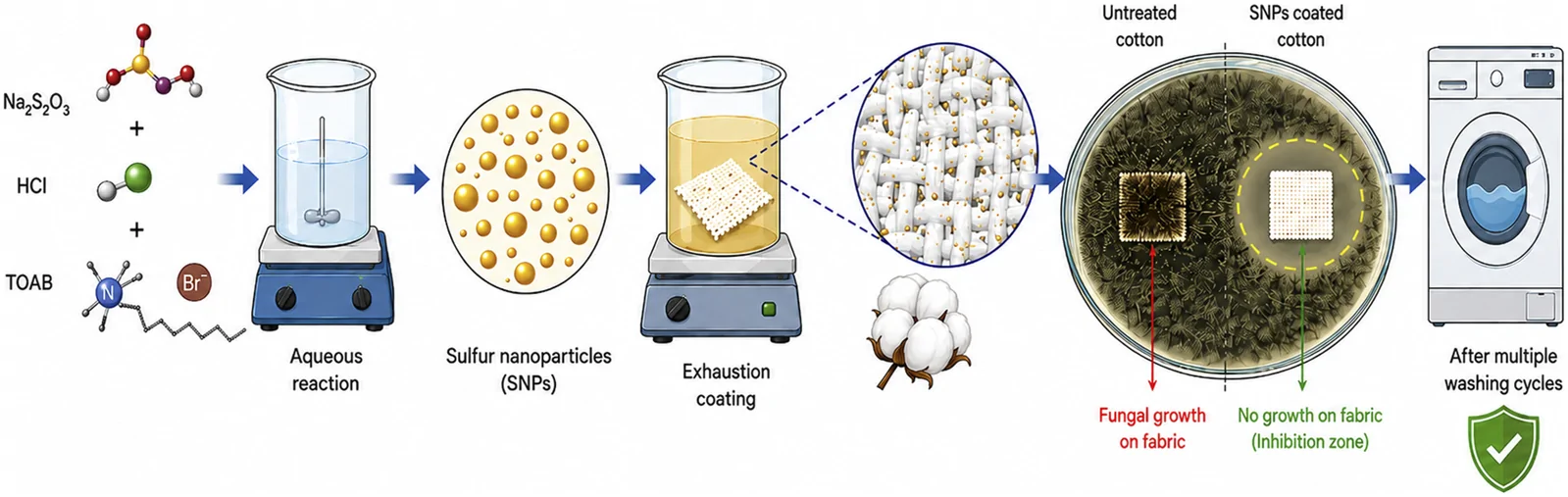

## 1 Introduction

Cotton is one of the most widely used natural fibres in the textile industry due to its softness, breathability, durability and biodegradability. These properties make it suitable for a wide range of applications, including clothing, home textiles and medical fabrics.^1^ However, the highly hydrophilic nature of cotton enables substantial moisture absorption, creating favourable conditions for fungal colonization, particularly under humid environments conditions.^2^ Fungal contamination of cotton fabrics can result in material deterioration through enzymatic degradation of cellulose, leading to discolouration, loss of mechanical strength and reduced service life.^3^ In addition, fungal growth on textiles may create hygiene concerns due to the release of airborne spores, allergens and mycotoxins, which can contribute to skin irritation and respiratory problems.^4^ Therefore, the development of durable antifungal cotton textiles is important for improving both material performance and user safety.

Various antifungal finishing approaches have been investigated for textile applications, including the incorporation of metal-based nanoparticles such as silver (Ag), copper (Cu) and zinc oxide (ZnO).^5–7^ Among these, silver nanoparticles (AgNPs) have received considerable attention due to their broad-spectrum antifungal activity and commercial applicability. However, their practical application is associated with several limitations, including high production cost, potential cytotoxicity, environmental persistence and nanoparticle release during laundering.^8–12^ Furthermore, increasing regulatory attention towards the environmental impact of metal-containing materials has necessitated the exploration for alternative antifungal agents. Similarly, conventional chemical antifungal finishes, such as quaternary ammonium compounds and triclosan, have raised concerns regarding toxicity, environmental accumulation, and reduced long-term effectiveness.^13^

These limitations further highlight the need for alternative antifungal materials that combine effective fungal control, durability, environmental compatibility, and ease of textile processing. Sulfur represents a promising candidate due to its long-established antifungal activity, low toxicity, abundance and cost-effectiveness.^14–17^ Recent advances in nanotechnology have enabled the conversion of bulk sulfur into sulfur nanoparticles (SNPs), which possess increased surface area, enhanced surface reactivity and improved antifungal activity compared with conventional sulfur.^14,18–20^ Previous studies have demonstrated that SNPs can inhibit fungal growth through multiple proposed mechanisms, including interaction with fungal cell membranes, disruption of cellular integrity and induction of oxidative stress.^18,21^

Although the antifungal activity of SNPs has been demonstrated in agricultural, biomedical, and related applications, their use in textile functionalization remains unexplored. To the best of our knowledge, no previous study has reported the use of SNPs specifically as an antifungal coating for cotton textiles. In particular, the incorporation of SNPs onto cotton fibres and the subsequent evaluation of their antifungal performance and retention of activity after repeated laundering have not been reported.

Nevertheless, achieving strong nanoparticle attachment to textile fibres remains a major challenge, as loosely bound nanoparticles may detach during laundering, resulting in a progressive loss of functionality.^22–24^ Several nanoparticle-based textile finishing approaches, including dip coating, exhaustion and pad-dry-cure processes, have been reported for incorporating functional nanoparticles onto fabrics.^22,23,25–30^ However, the suitability of these approaches for SNP-based cotton functionalization has not been comprehensively investigated. Therefore, in this study, we report the development of an antifungal coating for 100% cotton fabric using tetraoctylammonium bromide (TOAB)-stabilized SNPs. The SNP synthesis method was based on a previously reported chemical precipitation approach,^31^ whereas the present work focuses on their application to cotton textile functionalization using an exhaustion process and subsequent evaluation of nanoparticle deposition, physicochemical characteristics, antifungal activity against *Aspergillus niger* and retention of antifungal performance following repeated washing. The findings provide insight into the potential of SNPs as an alternative to conventional metal-based antifungal finishes for functional cotton textiles.

## 2 Experimental

### 2.1 Materials

Sodium thiosulfate pentahydrate (Na_2_S_2_O_3_·5H_2_O), hydrochloric acid (HCl, 32% w/v) and tetraoctylammonium bromide (TOAB) were used as received for the synthesis of SNPs. Distilled water, or sterile distilled water where appropriate, was used throughout the experiments. Plain woven 100% cotton fabric was used as the textile substrate. *Aspergillus niger* was selected as the test organism for antifungal evaluation in accordance with the American Association of Textile Chemists and Colorists (AATCC) TM 30 standard method for assessing the antifungal activity of textile materials. Potato dextrose agar (PDA) was purchased from Oxoid (UK), while mineral salts agar was obtained from HiMedia (India). Triton X-100 was used as a non-ionic wetting agent during antifungal testing. A haemocytometer (Marienfeld, Germany) was employed for fungal spore enumeration. Standard microbiological consumables, including sterile gauze, cotton swabs, glass spreaders and other aseptic laboratory equipment, were used throughout the microbiological experiments.

### 2.2 Sulfur nanoparticle (SNP) synthesis

SNPs were synthesized using a chemical precipitation method adapted from a previously reported procedure, with TOAB employed as a stabilizing agent to improve nanoparticle dispersion and minimize particle aggregation.^31^ An aqueous solution of sodium thiosulfate pentahydrate (Na_2_S_2_O_3_·5H_2_O, 0.80 M, 50.0 mL) was mixed with TOAB solution (0.02 M, 20.0 mL) under continuous stirring (120 rpm) at 40 °C. Hydrochloric acid (HCl, 1.0 M, 40.0 mL) was then added dropwise while maintaining constant stirring. The reaction mixture was stirred for 40 min, during which the formation of a yellow precipitate indicated the generation of elemental SNPs. The resulting SNPs were collected by centrifugation, washed repeatedly with distilled water to remove residual reactants and soluble impurities, and dried in a laboratory oven (BIOBASE). The dried nanoparticle powder was stored under ambient conditions until further characterization and textile coating experiments.

### 2.3 Characterization of synthesized SNPs

The formation and physicochemical properties of the synthesized SNPs were investigated using complementary spectroscopic, microscopic and colloidal characterization techniques. UV-vis-NIR spectrophotometer (Shimadzu UV-3600 Plus) was employed to confirm nanoparticle formation by analysing the optical absorption behaviour of SNP suspension over the wavelength range of 200–800 nm. The crystalline structure of the SNPs was analysed using an X-ray diffractometer (XRD) (Bruker D8 FOCUS model) equipped with a Cu anode. Cu Kα radiation with a wavelength of *λ* = 1.5406 Å was used, and the diffraction pattern was recorded in a 2*θ* locked-coupled configuration over a 2*θ* range of 5–90°. The surface morphology and particle characteristics were examined using field-emission scanning electron microscopy (FE-SEM, Hitachi SU6600) operated at an accelerating voltage of 20 kV. For SEM analysis, dried SNP powder samples were mounted onto conductive SEM stubs prior to imaging. The elemental composition of the synthesized nanoparticles was determined using energy-dispersive X-ray spectroscopy (EDS) coupled with AZtec software to confirm the presence of sulfur and associated elemental constituents. Fourier transform infrared (FTIR) spectroscopy (Bruker Vertex 80) was used to investigate the surface functional groups and possible interactions between SNPs and the stabilizing agent. Spectra were recorded in the range of 4000–400 cm^−1^ with a spectral resolution of 4 cm^−1^ and 32 scans per sample. The hydrodynamic particle size distribution, polydispersity index (PDI) and zeta potential of the SNP suspensions were determined using dynamic light scattering (DLS) analysis (Malvern particle size analyser) to evaluate particle dispersion characteristics and colloidal stability.

### 2.4 Anti-fungal activity of SNPs

The antifungal activity of SNPs was evaluated using the agar well diffusion method. Potato dextrose agar (PDA) was prepared by dissolving PDA powder (39 g L^−1^) in distilled water, followed by sterilization at 121 °C for 15 min. After cooling to 55–60 °C, the sterilized medium was aseptically poured into sterile Petri dishes and allowed to solidify. Wells were subsequently prepared on the agar using a sterile cork borer. An aliquot (10 µL) of SNP suspension was introduced into each well, and the plates were incubated at room temperature for 7 days. Antifungal activity was evaluated by measuring the diameter of the inhibition zone formed around each well. The obtained inhibition zones were used to confirm the antifungal activity of SNPs prior to their application onto cotton fabric substrates.

### 2.5 Coating of cotton fabric with SNPs

The synthesized SNPs were applied to cotton fabric using an exhaustion method, a widely employed approach for the incorporation of functional nanoparticles into textile substrates.^32,33^ Woven 100% cotton fabric was cut into specimens (5 × 5 cm) and pre-treated by scouring in distilled water at a material-to-liquor ratio (MLR) of 1 : 20 for 20 min at 50–70 °C to remove surface impurities and any residual textile finishing agents. The pre-treated fabrics were then immersed in an aqueous SNP dispersion at an MLR of 1 : 10 containing different SNP concentrations (up to 5% on the weight of fabric, owf). The treatment was carried out at 50 °C for 30 min under continuous agitation to facilitate uniform nanoparticle uptake and distribution throughout the fabric surface. After treatment, excess dispersion was removed, and the fabrics were dried at 80 °C for 20 min in a drying oven (BIOBASE). The drying process was employed to promote the retention of SNPs on the cotton fibre surface. The treated fabrics were stored under dry, dust-free conditions prior to further physicochemical characterization and antifungal evaluation.

### 2.6 Characterization of coated fabric

Both unwashed and washed SNP-treated cotton fabrics were characterized to evaluate nanoparticle deposition, surface morphology, elemental composition and possible interactions between the coating and cotton substrate. The surface morphology of untreated, SNP-coated and washed SNP-coated fabrics was examined using field emission scanning electron microscopy (FE-SEM, Hitachi SU6600). Prior to imaging, fabric samples were sputter-coated with a thin layer of gold to improve electrical conductivity. SEM images were acquired using a backscattered electron detector at an accelerating voltage of 20 kV, and different magnifications were used to evaluate nanoparticle distribution on the fibre surface. The elemental composition of the coated fabrics was analysed using energy-dispersive X-ray spectroscopy (EDS, Oxford Instruments) equipped with AZtec software. Measurements were performed at an accelerating voltage of 20 kV and a working distance of approximately 15 mm to confirm the presence and retention of sulfur on the cotton surface before and after washing. Diffuse reflectance UV-vis spectra of unwashed SNP-treated and washed SNP-treated fabrics were recorded using a UV-vis spectrophotometer equipped with an integrating sphere (Shimadzu UV-3600 Plus) to assess changes in optical properties associated with nanoparticle deposition and coating durability. Spectra were collected over the wavelength range of 200–800 nm with a sampling interval of 0.5 nm. Each spectrum represents the average of three scans, and untreated cotton fabric was used as the baseline reference. Fourier transform infrared (FTIR) spectroscopy was performed using a Bruker Vertex 80 FTIR spectrometer to investigate possible chemical interactions between the SNP coating and the cotton fibres. Spectra were recorded in the range of 4000–400 cm^−1^ at a resolution of 4 cm^−1^ with 32 scans per sample.

### 2.7 Washing durability test

The washing durability and retention behaviour of SNP coatings on cotton fabrics were evaluated through repeated laundering cycles under controlled conditions. Fabric samples were subjected to washing cycles using a front-loading washing machine (Singer) at selected intervals (0, 5 and 10 washing cycles). Each washing cycle was carried out for 30 min at 40 °C, followed by rinsing with distilled water and drying at 80 °C for 20 min using a drying oven (BIOBASE). Washing experiments were conducted both in the presence and absence of surfactant to evaluate the influence of laundering conditions on coating stability. However, subsequent durability evaluations were performed without surfactant to minimize interference coming from the antimicrobial agents of commercial detergents during the assessment of the intrinsic antifungal performance of the SNP coating. After 5 and 10 washing cycles, treated fabrics were evaluated using scanning electron microscopy (SEM), energy-dispersive X-ray spectroscopy (EDS), UV-vis diffuse reflectance analysis and antifungal testing to investigate nanoparticle retention, surface modification and preservation of antifungal activity compared with unwashed coated fabrics.

### 2.8 Antifungal activity of coated fabric

The antifungal activity of SNP-coated cotton fabrics against *Aspergillus niger* was evaluated according to the AATCC TM 30 standard test method for antifungal activity of textile materials (Test III: Agar Plate Method).^34^ Fabric specimens (1 × 1 cm) were prepared from both untreated and SNP-treated cotton samples for antifungal evaluation. A spore suspension of *A. niger* was prepared from a 14 days-old culture grown on PDA. Sterile distilled water (10 mL) was added to the culture, and the spores were gently scraped from the culture surface using a sterile glass spreader. The suspension was gently mixed to obtain a homogeneous spore dispersion minimizing disruption of fungal structures. The resulting suspension was transferred into a flask containing 50 mL of sterile distilled water and filtered through sterile gauze to remove residual mycelial fragments and debris. The spore concentration was determined using a haemocytometer and adjusted to approximately 1.0 × 10^6^ CFU mL^−1^.

Mineral salts agar was used as the test medium. The prepared inoculum (0.5 ± 0.1 mL) was uniformly distributed onto the agar surface. Prior to testing, Fabric specimens were pre-wetted in sterile water containing 0.05% Triton X-100 to improve moisture penetration and fungal contact.^35^ The specimens were then placed onto the inoculated agar surface, followed by the application of additional inoculum (0.2 ± 0.01 mL) onto each fabric specimen. Untreated cotton fabric was used as a negative control to confirm fungal viability and to evaluate the inherent susceptibility of cotton to fungal colonization. All samples were incubated at 28 ± 1 °C for 14 days under aerobic conditions. Antifungal performance was evaluated by observing fungal growth on and around the fabric specimens. The presence or absence of fungal colonization was recorded, and the inhibition zone diameter (mm) was measured where applicable.

### 2.9 Statistical analysis

All experiments related to antifungal performance were performed in triplicate, and the results are presented as mean values with standard deviation (SD). Statistical analysis was conducted using Origin 2026 software to evaluate variations among untreated cotton fabrics, SNP-treated fabrics and washed SNP-treated fabrics. The mean inhibition zone values and corresponding SD values were calculated to assess the reproducibility of the antifungal performance and the influence of SNP treatment and washing cycles on coating effectiveness.

## 3 Results and discussion

### 3.1 Confirmation of SNPs formation

The formation of SNPs during the synthesis process was initially indicated by a visible colour change in the reaction mixture. The initially colourless sodium thiosulfate solution gradually became turbid and developed a characteristic yellow colouration (Fig. S1, SI), suggesting the formation of elemental sulfur particles through the acid-induced decomposition of thiosulfate ions. This visual observation indicates the occurrence of sulfur nucleation and precipitation within the reaction medium in the presence of TOAB as a stabilizing agent.^31^

Following synthesis, the formed SNPs were separated by centrifugation and repeatedly washed with deionized water to remove unreacted precursors and soluble reaction by-products. The purified product was obtained as a stable yellow powder after drying. The formation and physicochemical characteristics of the synthesized SNPs were subsequently confirmed through complementary characterization techniques, including UV-vis spectroscopy, XRD analysis, DLS, zeta potential analysis, FTIR spectroscopy, SEM and EDS analysis, as discussed in the following sections.

### 3.2 Characterization of synthesized SNPs

#### 3.2.1 UV-vis analysis

**Fig. 1:**
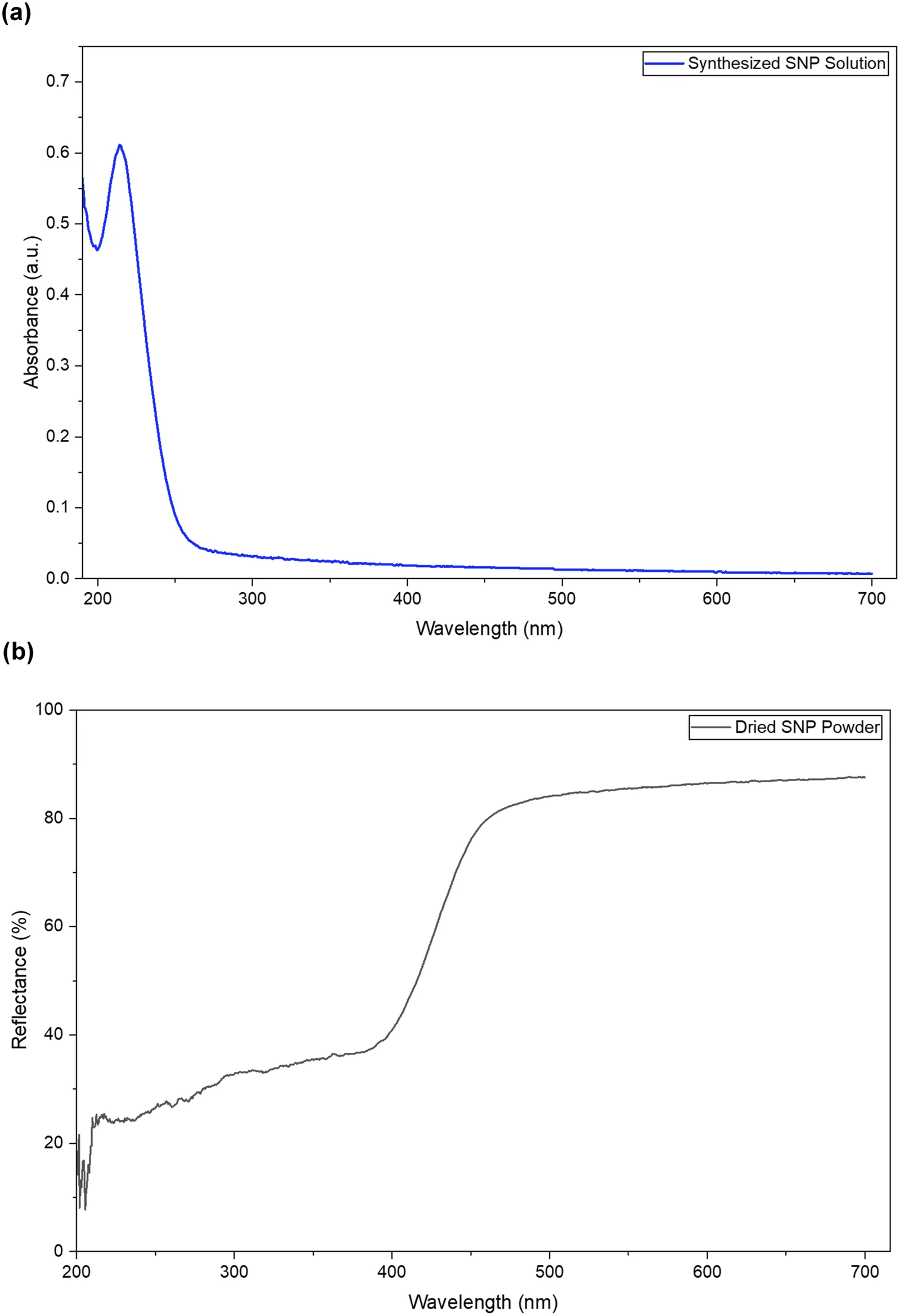
Optical characterization of synthesized SNPs (a) UV-vis absorption spectrum of SNP solution and (b) UV-visible diffuse reflectance spectrum of dried SNPs.

The optical properties of the synthesized SNPs were investigated using UV-vis spectroscopy over the wavelength range of 200–800 nm. As shown in Fig. 1, the TOAB-stabilized SNP suspension exhibited a characteristic absorption peak at approximately 220 nm. This absorption feature is consistent with previously reported SNPs, for which absorption maxima around 220–225 nm have been attributed to SNP formation.^36^ The observed peak confirms the successful generation of SNPs during the precipitation process.

To further evaluate the optical characteristics of the synthesized material, diffuse reflectance UV-vis spectroscopy was performed on the dried SNP powder (Fig. 1b). A decrease in reflectance was observed from approximately 430 nm, corresponding to the absorption edge of SNPs. Compared with bulk sulfur, which exhibits optical absorption at longer wavelengths (∼520 nm), the observed shift toward shorter wavelengths indicates changes in optical behaviour associated with particle size reduction and nanoscale effects.^37^ Similar blue shifts have been reported for SNP systems due to alterations in electronic properties arising from reduced particle dimensions.

The UV-vis results confirm the formation of SNPs, while further structural and size-related characteristics were evaluated using XRD, DLS, SEM and FTIR analyses.

#### 3.2.2 X-ray diffraction analysis of synthesized SNPs

**Fig. 2:**
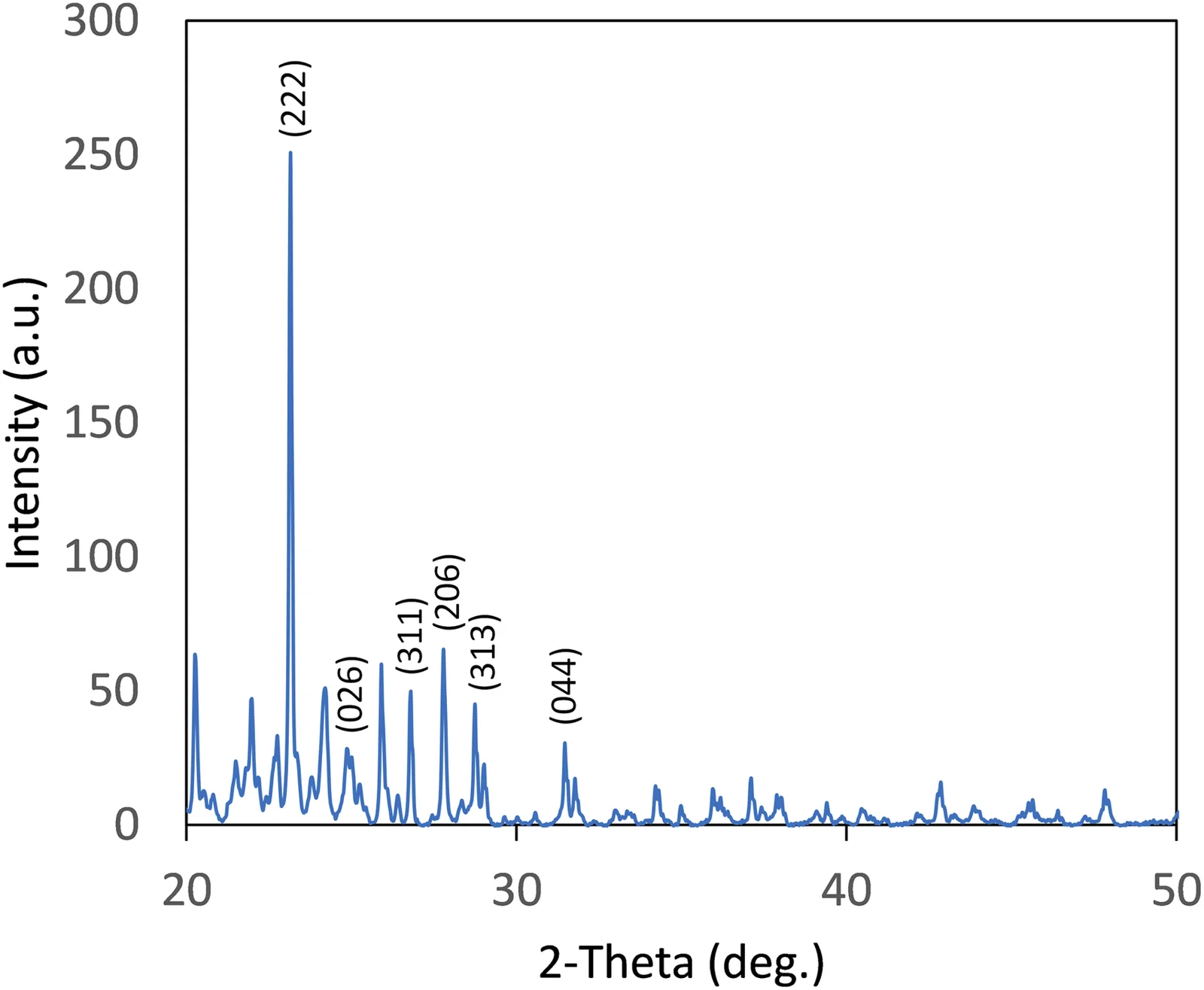
XRD pattern of synthesized SNPs.

The XRD pattern of the synthesized SNPs showed several distinct diffraction peaks (Fig. 2), indicating that the prepared sulfur was predominantly crystalline.^38^

The most prominent reflection was observed at 2*θ* = 23.16°, while other prominent reflections occurred at approximately 25.94°, 26.80°, 27.79°, 28.75° and 31.49°. Additional weaker reflections were observed at higher diffraction angles. This resulted XRD pattern shows several characteristic diffraction peaks corresponding to crystalline elemental sulfur, confirming the growth of a crystalline sulfur phase.^38^ Similar SNP studies have reported characteristic reflections in the 23–32° region, supporting the assignment to orthorhombic sulfur.^38–42^ The presence of multiple well-defined diffraction peaks, rather than a broad amorphous halo, further confirms the formation of crystalline elemental sulfur.

The nanoscale of the synthesized material was further studied through crystallite-size estimation using the Scherrer equation,^43^*D* = 0.9*λ*/*β* cos *θ*where (*D*) is the apparent mean crystallite size, (K) is the shape factor (0.9), *λ* is the Cu Kα wavelength (0.15406 nm), *β* is the full width at half maximum (FWHM) of the diffraction peak in radians, and *θ* is the Bragg angle. The estimated crystallite sizes from the principal sulfur reflections were approximately 57–74 nm, with an average value of about 66 nm. These values represent the apparent dimensions of coherent crystalline domains rather than the overall hydrodynamic particle size.

#### 3.2.3 Size distribution and stability of sulfur suspension

Dynamic light scattering (DLS) analysis was performed to evaluate the hydrodynamic particle size distribution and colloidal stability of the synthesized SNP suspension. The measured particle size was above 100 nm, which is expected for DLS measurements because this technique determines the hydrodynamic diameter rather than the physical core size of individual nanoparticles. The measured value includes contributions from the SNP core, surface-associated TOAB molecules, surrounding solvent layers and any weakly associated nanoparticle aggregates present in the dispersion.^44^ Therefore, the hydrodynamic diameter obtained from DLS is generally larger than the actual nanoparticle core size determined by direct imaging techniques.

**Fig. 3:**
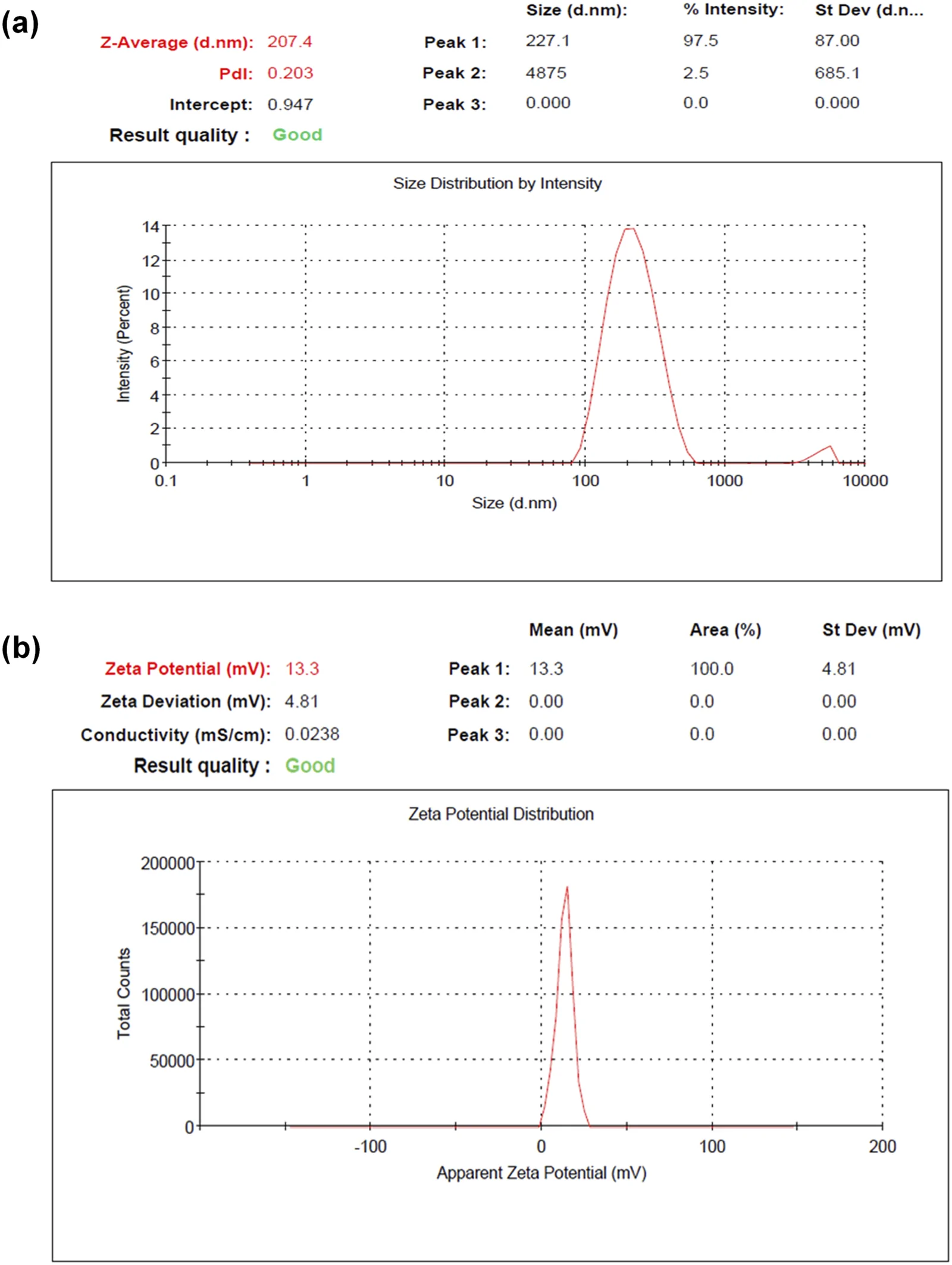
Particle size and surface charge analysis of synthesized SNPs (a) particle size distribution of SNPs determined by DLS and (b) apparent zeta potential distribution of synthesized SNPs.

The intensity-based particle size distribution (Fig. 3a) showed a dominant particle population with a minor secondary peak at larger diameters, indicating the presence of a small fraction of aggregated particles or particle assemblies with different surface interactions.^45^ Such behaviour is commonly observed in nanoparticle suspensions due to the high surface energy of nanoscale materials and their tendency to undergo partial aggregation.^45^

The TOAB-stabilized SNP suspension exhibited an average hydrodynamic diameter of approximately 207 nm. The observed particle size can be associated with the dual stabilization effect provided by TOAB. The quaternary ammonium head group may interact with the SNP surface, providing electrostatic stabilization, while the long hydrophobic alkyl chains may contribute steric effects that reduce excessive particle aggregation.^46^ However, the presence of surfactant molecules and loosely associated nanoparticle assemblies can increase the apparent hydrodynamic diameter measured by DLS.^30^ The polydispersity index (PDI) was used to assess the uniformity of the particle size distribution. Although higher PDI values (>0.7) generally indicate highly heterogeneous nanoparticle systems, the synthesized SNP suspension exhibited PDI values in the range of 0.2–0.3, indicating a relatively narrow particle size distribution and acceptable dispersion uniformity.^47^ These results suggest that TOAB effectively controlled uncontrolled aggregation during nanoparticle formation.

The surface charge and colloidal stability of SNPs were further evaluated through zeta potential measurements. Zeta potential is influenced by factors including surface functional groups, stabilizing molecules, pH and ionic strength of the dispersion medium.^48^ Generally, higher absolute zeta potential values (>20–30 mV) indicate stronger electrostatic repulsion and improved colloidal stability, whereas lower values may allow partial aggregation.^49^ As shown in Fig. 3b, TOAB-stabilized SNPs exhibited a positive zeta potential of +13.3 mV, which can be attributed to the adsorption of positively charged quaternary ammonium groups of TOAB onto the SNP surface. Although the measured zeta potential indicates moderate electrostatic stabilization rather than highly stable electrostatic repulsion, the relatively low PDI value and controlled particle size distribution demonstrate that TOAB contributes to maintaining a reasonably dispersed SNP system. The combined DLS, PDI and zeta potential results confirm successful formation of TOAB-stabilized SNPs with controlled dispersion characteristics suitable for subsequent textile coating applications.

#### 3.2.4 Surface morphology and elemental composition of SNPs

SEM analysis was performed to investigate the morphology and surface characteristics of the synthesized SNPs prepared using TOAB as a stabilizing agent. The SEM images presented in Fig. S2 (SI) reveal that the synthesized particles possess an irregular morphology with no well-defined morphology. At higher magnification, the nanoparticles appear as aggregated spherical domains with relatively smooth surfaces and localized rough regions. The observed aggregation is commonly associated with the high surface energy of SNPs and strong interparticle interactions during drying and sample preparation.^41^ Similar agglomeration behaviour has been reported for SNPs synthesized using surfactant-assisted precipitation methods, where nanoparticle aggregation occurs despite the presence of stabilizing agents.^41^

The elemental composition of the synthesized nanoparticles was further examined using SEM coupled with energy-dispersive X-ray spectroscopy (SEM-EDS) (Fig. S3, SI). A strong sulfur signal was detected, confirming the formation of elemental SNPs. The presence of carbon can be attributed primarily to the TOAB stabilizing molecules adsorbed on the nanoparticle surface as well as the carbon conductive tape used during SEM sample preparation. The weak oxygen signal may originate from surface-adsorbed moisture or minor surface oxidation during sample handling.^50^

#### 3.2.5 FTIR analysis of SNPs

**Fig. 4:**
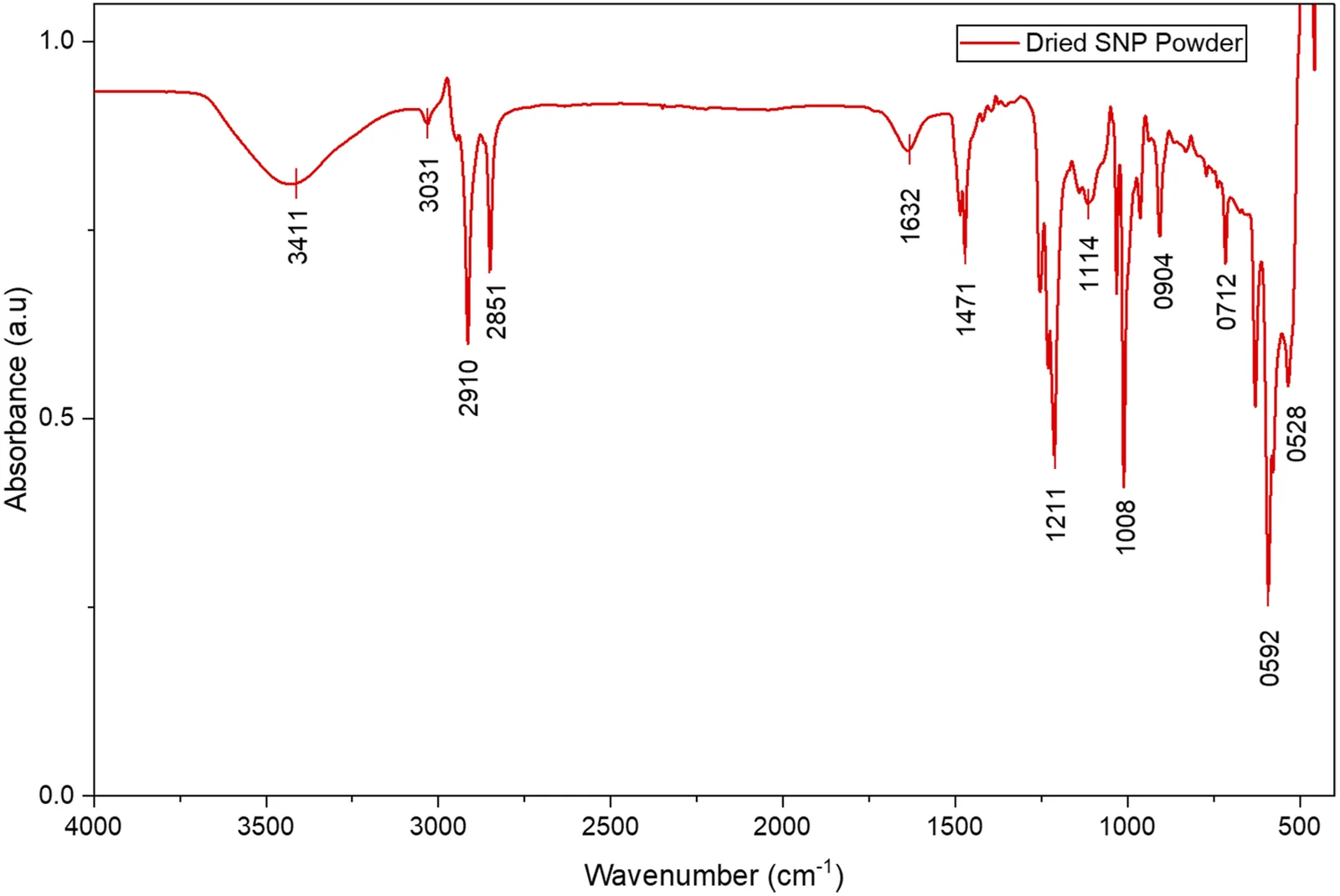
Infrared spectrum of the SNPs.

FTIR spectroscopy was employed to investigate the surface functional groups associated with the synthesized TOAB-stabilized SNPs and to confirm the presence of surface-bound stabilizing molecules. The FTIR spectrum of TOAB-stabilized SNPs is presented in Fig. 4.

A broad absorption band centred at 3411 cm^−1^ is attributed to O–H stretching vibrations, which can be assigned to adsorbed moisture and/or surface hydroxyl groups formed during nanoparticle processing.^51^ The characteristic absorption bands observed at 3031, 2910 and 2851 cm^−1^ correspond to C–H stretching vibrations of the long alkyl chains of TOAB, indicating the presence of the surfactant associated with the nanoparticle surface.^51–53^ The bending vibrations of CH_2_ and CH_3_ groups appearing in the range of 1470–1380 cm^−1^ further support the contribution of the alkyl chains originating from TOAB. The absorption band at 1211 cm^−1^ can be assigned to C–N stretching vibrations associated with the quaternary ammonium group of TOAB, while the bands observed in the 1114–1008 cm^−1^ region are attributed to additional C–N/C–O related vibrations of the stabilizing molecule.^51–53^ In the low-wavenumber region, weak absorption bands around 592 and 528 cm^−1^ are associated with S–S stretching vibrations, confirming the presence of sulfur species in the synthesized nanoparticles.^54^

The simultaneous observation of sulfur-related vibrations and TOAB characteristic functional groups confirm the successful association of TOAB with the SNP surface and it's role as a surface-capping agent.^55^

#### 3.2.6 Antifungal activity of SNPs

**Fig. 5:**
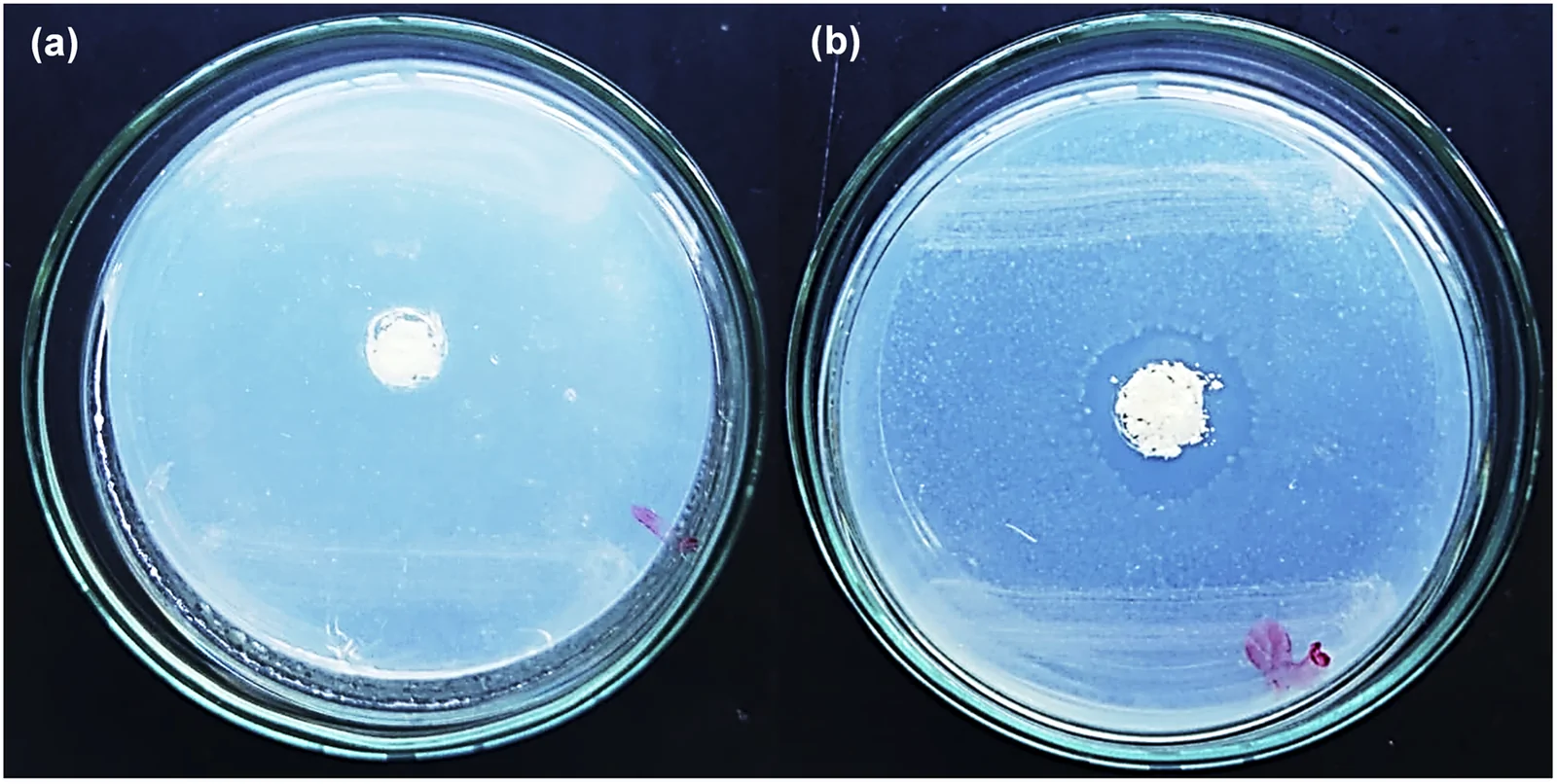
Antifungal activity of (a) bulk sulfur and (b) synthesized SNPs.

The antifungal performance of the synthesized SNPs was initially evaluated to confirm their suitability as an active antifungal component for textile functionalization. Although SNPs have been reported to exhibit antimicrobial properties, investigations specifically focusing on their antifungal activity and potential application in textile systems remain limited.^18,21^ In the present study, TOAB-stabilized SNPs demonstrated noticeable inhibitory activity against the tested fungal strains (Fig. 5b), confirming their potential as a functional antifungal material.

The antifungal activity of SNPs is generally associated with their nanoscale characteristics and surface interactions with fungal cells. Although the exact mechanism has not yet been fully established, previous studies have proposed several possible pathways, including adsorption of nanoparticles onto the fungal cell surface, disruption of membrane integrity, leakage of intracellular components and subsequent inhibition of cellular functions.^18^ In addition, sulfur-containing reactive species generated from nanoscale sulfur may contribute to oxidative stress, further affecting fungal metabolism and viability.^21^

The enhanced antifungal behaviour of TOAB-stabilized SNPs may also be influenced by surface modification provided by the cationic stabilizer. TOAB adsorption onto SNPs introduces positively charged quaternary ammonium groups on the particle surface, which may promote electrostatic interactions with negatively charged fungal cell wall components, including acidic polysaccharides and phosphorylated molecules. Such interactions could facilitate closer contact between SNPs and fungal cells, thereby enhancing membrane-associated damage and antifungal activity.^56,57^ Therefore, the observed antifungal activity in the present study is likely related to the combined effects of nanoscale sulfur, increased surface reactivity and interaction between SNPs and fungal cells.

Furthermore, bulk sulfur without nanoscale modification showed comparatively lower antifungal activity under the tested conditions (Fig. 5a), suggesting that particle size reduction and surface stabilization play important roles in improving sulfur-based antifungal performance. This observation agrees with previous reports demonstrating enhanced antimicrobial activity of SNPs compared with conventional elemental sulfur due to their increased surface area and reactivity.^18^

These findings confirm that TOAB-stabilized SNPs possess antifungal activity and demonstrate their suitability as an active component for developing metal-free antifungal textile coatings.

### 3.3 Characterization of treated fabrics

#### 3.3.1 Surface morphology and chemical composition of treated fabrics

**Fig. 6:**
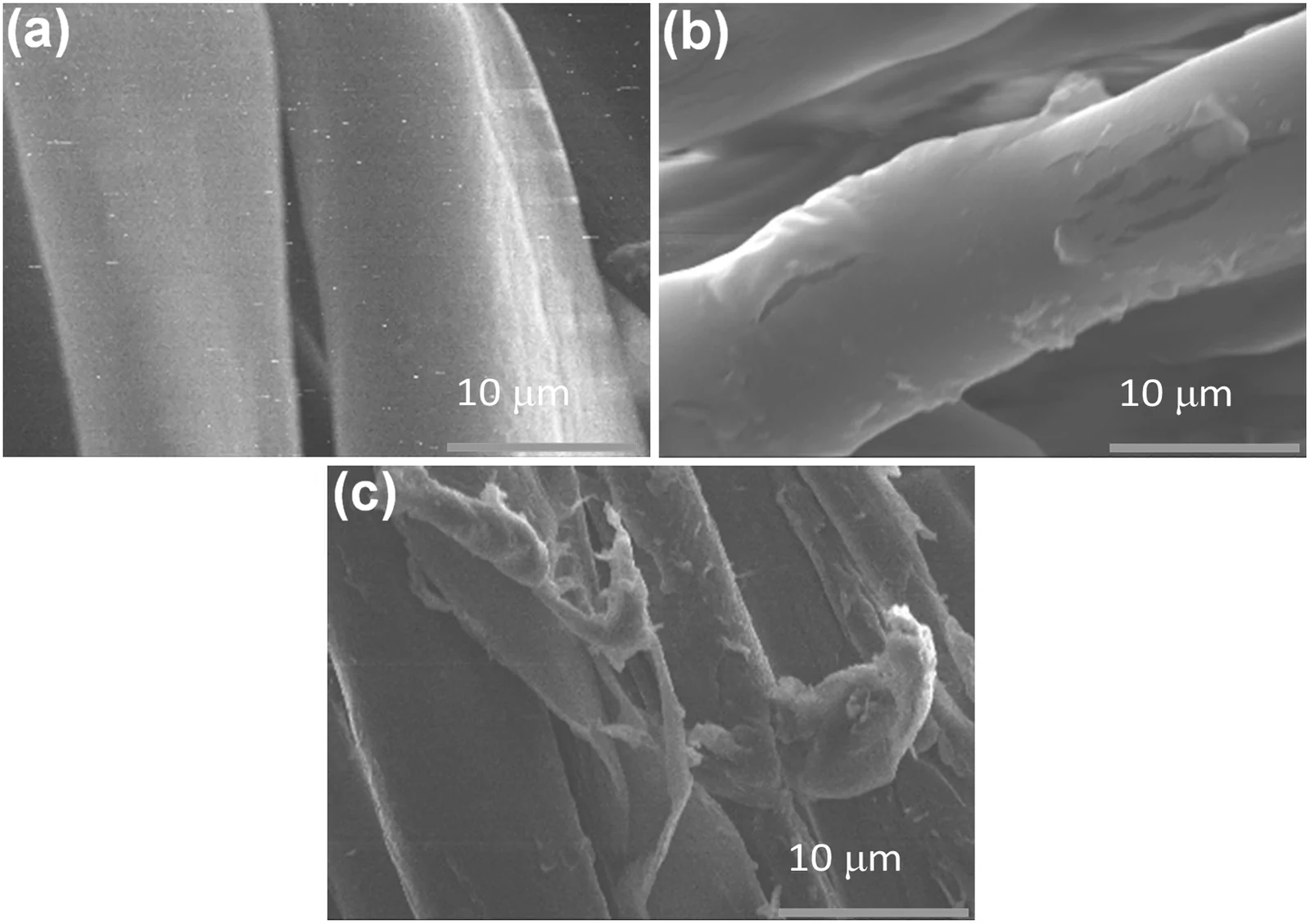
SEM micrographs of cotton fabrics (a) untreated cotton fabric with smooth fibre morphology, (b) SNP-coated cotton fabric showing nanoparticle deposition on the fibre surface and (c) treated fabric after 10 washing cycles.

SEM was employed to investigate the surface morphology and nanoparticle deposition behaviour of SNP-treated of cotton fabrics treated with SNPs (5.0% owf). The untreated cotton fabric exhibited a relatively smooth fibre surface with characteristic longitudinal grooves associated with the cellulose structure, and no visible particulate deposits were observed (Fig. 6a). In contrast, SNP-treated cotton fibres showed noticeable surface modification, with fine particulate deposits distributed along the fibre surface (Fig. 6b). Different magnification SEM images (Fig. S4, SI) further revealed the presence of nanoscale particulate structures attached to the fibre surface, providing additional evidence for successful SNP deposition.

Following 10 washing cycles, the overall morphology of the cotton fibres remained preserved, and residual particulate deposits were still observed on the fibre surface (Fig. 6c). Although a reduction in deposited particles was evident after washing, the remaining surface-associated particles indicate that a fraction of SNPs remained attached to the cotton fibres. The slight increase in apparent particle size compared with the originally synthesized SNPs may be attributed to nanoparticle aggregation during the exhaustion treatment and interactions between nanoparticles and the cellulose fibre surface.^58^

**Fig. 7:**
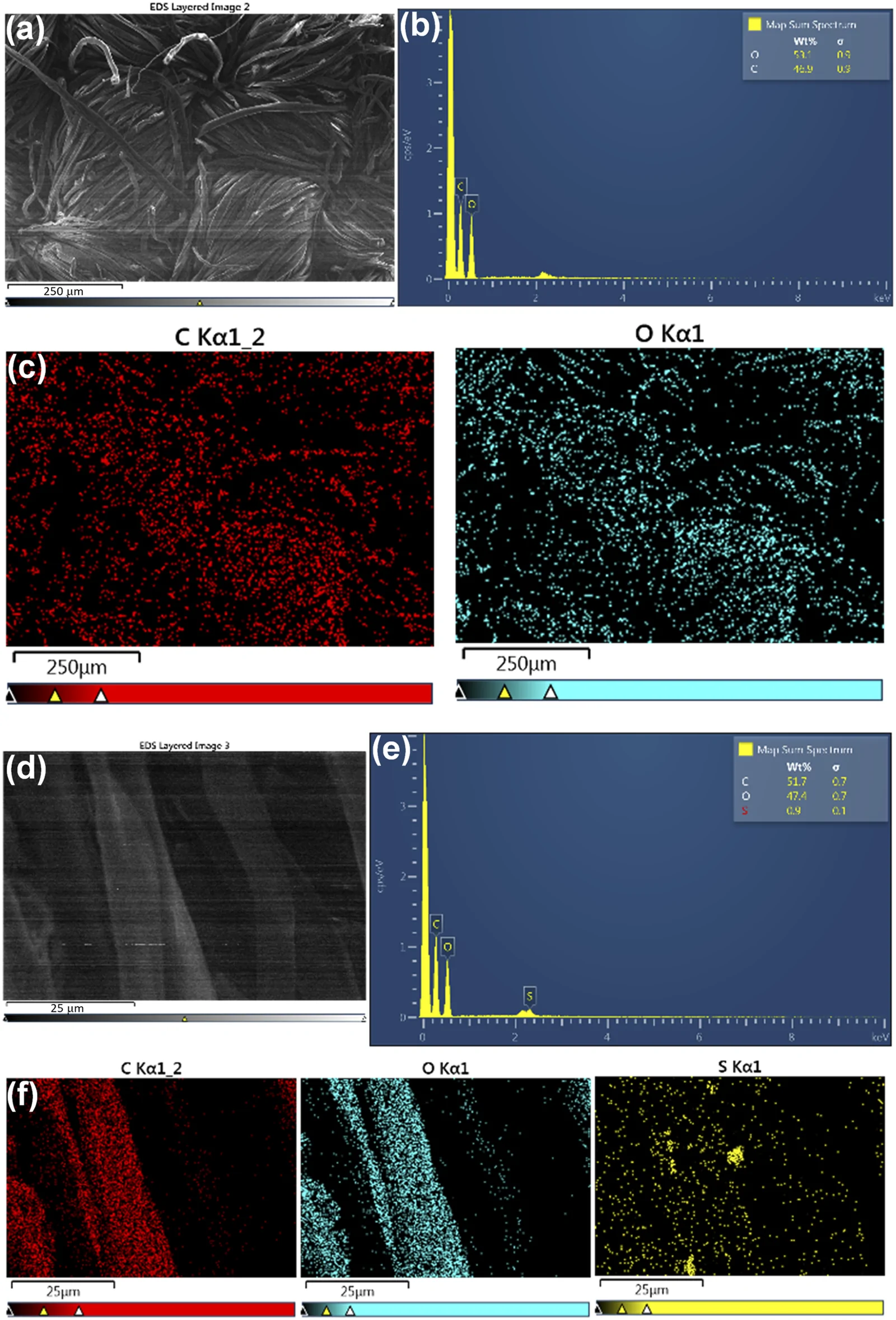
SEM-EDS analysis of untreated and SNP-treated cotton fabrics (a)–(c) SEM image, EDS spectrum and elemental maps of the untreated fabric, respectively and (d)–(f) corresponding SEM image, EDS spectrum and elemental maps of the SNP-treated fabric, showing the distribution of carbon (red), oxygen (blue) and sulfur (yellow).

The elemental composition and spatial distribution of the deposited SNPs were analysed using SEM-EDS and elemental mapping. As shown in Fig. 7b, untreated cotton fabric exhibited only carbon and oxygen signals, corresponding to the cellulose composition, with no detectable sulfur contribution. In comparison, sulfur was clearly detected in the EDS spectrum of the SNP-treated cotton fabric (Fig. 7e), confirming the presence of SNPs on the fibre surface. Carbon and oxygen remained the predominant elements due to the cellulose-based nature of cotton, while the relatively lower sulfur intensity can be attributed to the low loading and surface-localized distribution of SNPs.

The corresponding elemental mapping further showed the spatial distribution of sulfur across the observed fibre surfaces, providing additional evidence for the deposition of SNPs onto the cotton fibres. The distribution of sulfur signals, together with the particulate features observed in the SEM images, supports the successful incorporation of SNPs during the exhaustion treatment.

**Fig. 8:**
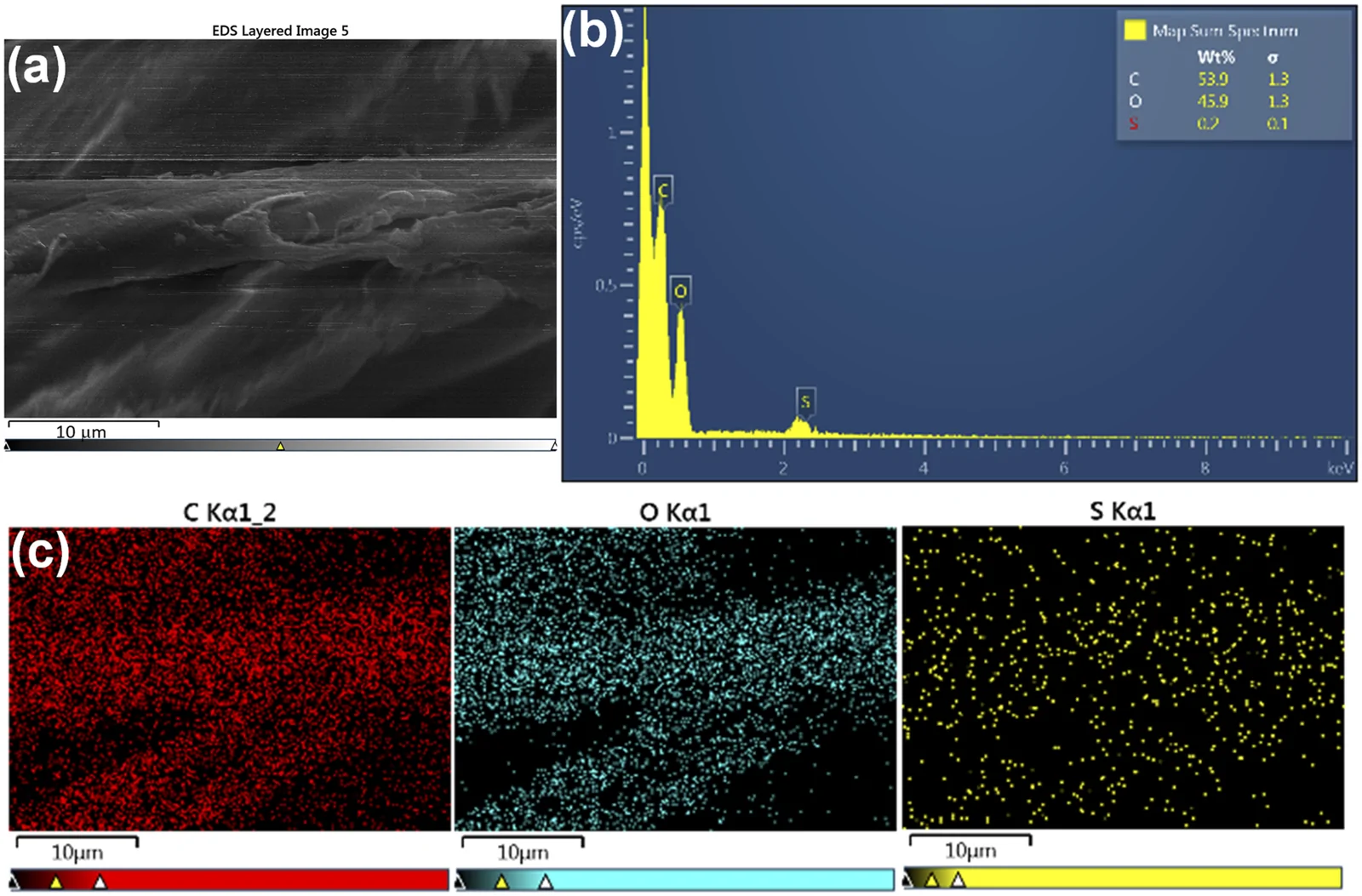
SEM-EDS analysis and elemental mapping of 10 times washed treated cotton fabrics (a) SEM image, (b) EDS spectra and (c) elemental maps showing carbon (red), oxygen (blue) and sulfur (yellow).

After 10 washing cycles, sulfur was still detected in the treated fabric samples (Fig. 8), although with reduced signal intensity compared with the unwashed sample. The persistence of sulfur even after laundering indicates that a proportion of the deposited SNPs remained associated with the cotton substrate, whereas the reduction in signal intensity suggests partial removal of loosely attached SNPs during washing. The corresponding elemental mapping also confirmed the continued presence of sulfur on the fibre surface after washing. Additional SEM images and corresponding elemental maps obtained from different regions of the fabric before and after washing are provided in the SI (Fig. S5 and S6 SI), providing further evidence of the reproducibility of sulfur deposition and its retention after laundering.

The combined SEM morphology, EDS elemental analysis and sulfur mapping provide complementary evidence for successful deposition and partial retention of SNPs on cotton fibres after repeated laundering.

#### 3.3.2 UV-vis analysis of treated fabrics

**Fig. 9:**
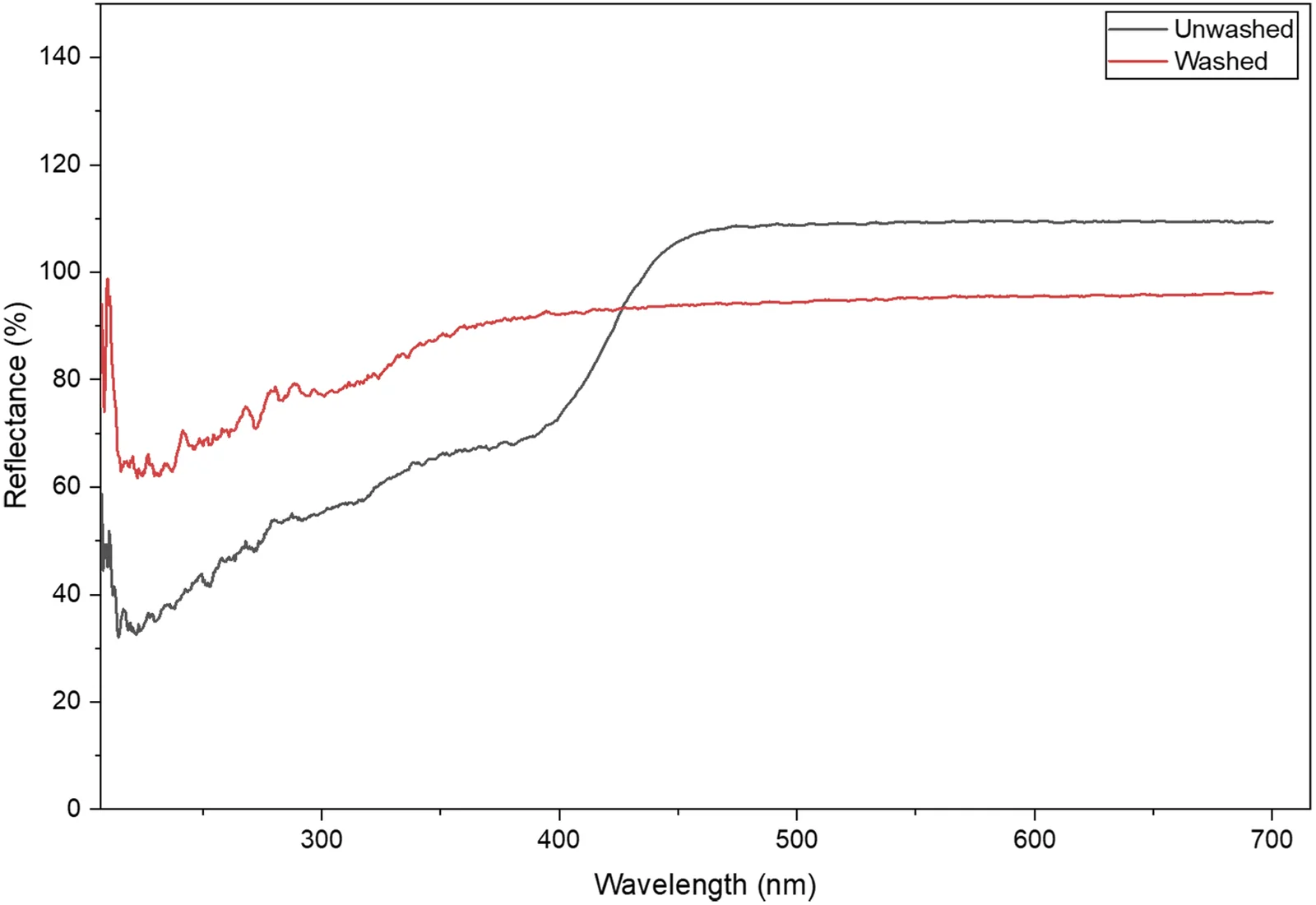
Solid-state UV-vis spectra of synthesized SNPs treated fabric before washing and after 10 washing cycles.

Diffuse reflectance UV-vis spectroscopy was employed to evaluate the optical response of SNP-treated cotton fabrics and to investigate changes in coating retention before and after repeated washing (Fig. 9). Significant differences were observed in the UV region (200–300 nm) between unwashed and washed SNP-treated fabrics. The unwashed SNP-treated fabric exhibited reduced reflectance compared with untreated cotton, indicating enhanced UV absorption associated with the presence of SNPs.

Following washing, a partial increase in reflectance was observed, which can be attributed to the removal of loosely attached nanoparticles or redistribution of surface-deposited SNPs during laundering. However, the reflectance values remained lower than those of untreated cotton, indicating that a considerable proportion of SNPs remained associated with the fibre surface after washing. Similar changes in optical response have been reported as an indication of nanoparticle retention on textile substrates after laundering.^59^

After 10 washing cycles, the treated fabric maintained a comparable spectral profile, suggesting reasonable stability of the SNP coating under the applied washing conditions. The retained UV absorption behaviour, together with SEM and EDS observations, indicates that SNPs were not completely removed during laundering. The absence of significant changes in the visible region further suggests that SNP treatment does not substantially alter the appearance of cotton fabrics, which is advantageous for practical textile applications. Therefore, diffuse reflectance UV-vis analysis provides additional evidence supporting successful SNP deposition and partial retention of the coating after repeated washing.

#### 3.3.3 FTIR analysis of treated fabrics

**Fig. 10:**
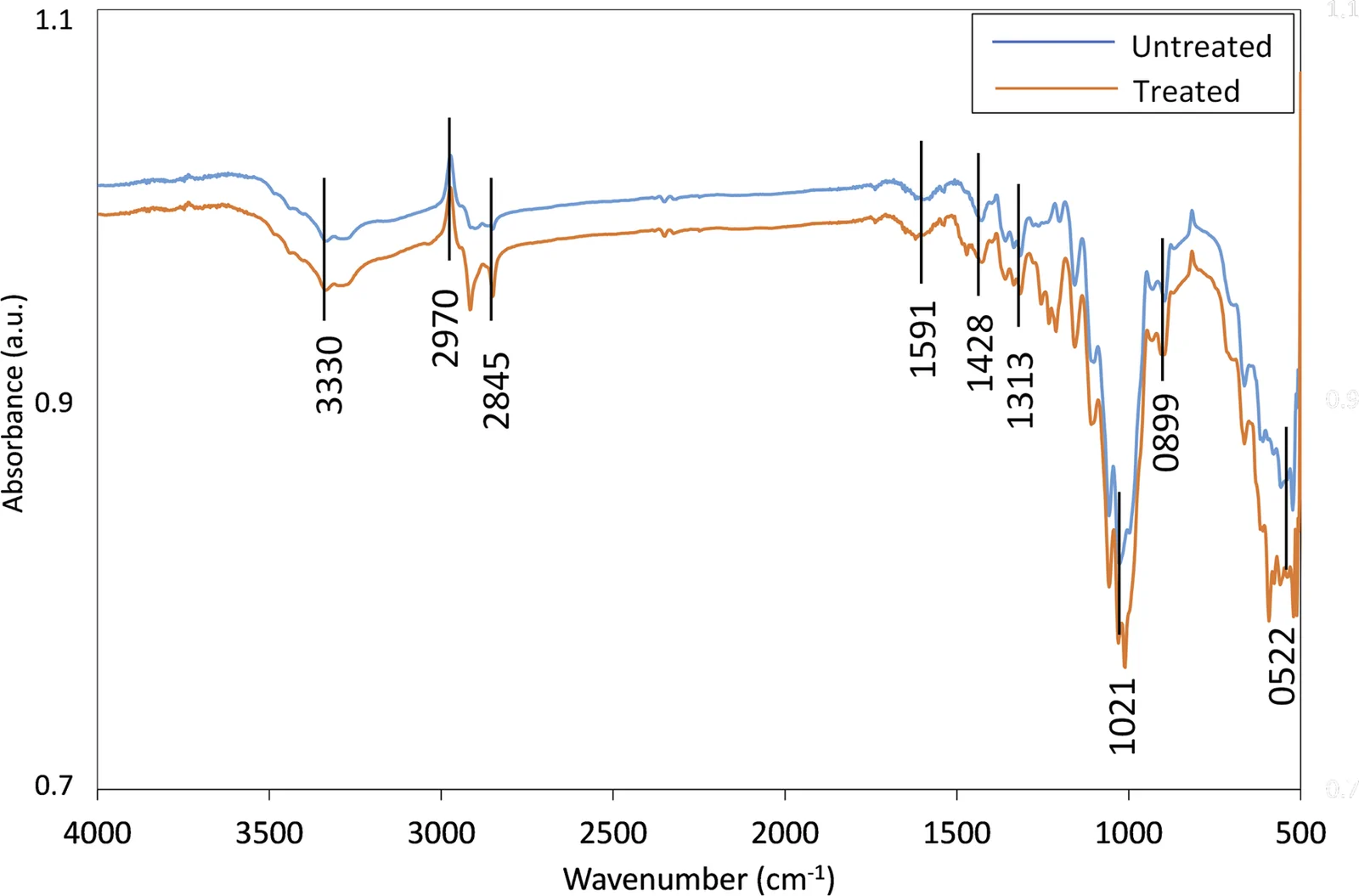
FTIR spectrum of untreated 100% cotton woven fabric and treated fabric.

FTIR spectroscopy was performed to investigate possible chemical changes in cotton fabrics following SNP treatment and to evaluate the interaction between deposited SNPs and the cellulose substrate (Fig. 10).

The FTIR spectrum of untreated cotton fabric exhibited the characteristic absorption bands associated with cellulose. A broad band centred at approximately 3330 cm^−1^ corresponds to O–H stretching vibrations originating from intra- and intermolecular hydrogen bonding within cellulose chains.^60^ The absorption bands observed at 2970 and 2845 cm^−1^ are attributed to C–H stretching vibrations of aliphatic –CH and –CH_2_ groups.^61^ The band at 1591 cm^−1^ corresponds to H–O–H bending vibrations associated with absorbed moisture, while the peak at 1428 cm^−1^ is related to CH_2_ scissoring vibrations of crystalline cellulose regions. Additional cellulose-associated bands were observed 1313 cm^−1^ (O–H bending), 1021 cm^−1^ (C–O–C and C–O stretching of the polysaccharide backbone) and 899 cm^−1^ (β-glycosidic linkages vibrations), confirming the presence of cellulose I structure.^62^

After SNP treatment, the FTIR spectrum of cotton fabric retained the characteristic cellulose absorption bands without significant peak shifts or the appearance of new strong absorption bands (Fig. 10b). This indicates that the fundamental chemical structure of cellulose was preserved during the coating process. However, slight changes in band intensity and minor broadening, particularly in the hydroxyl stretching region and fingerprint region, were observed after SNP treatment. These variations may result from interactions between surface-bound TOAB-stabilized SNPs and hydroxyl groups present on cellulose fibres.^53^ The absence of distinct sulfur-related bands can be explained by the relatively low concentration and surface-localized distribution of SNPs, together with the overlap of weak sulfur vibrations with the dominant cellulose absorption bands. Similar behaviour has been reported for nanoparticle-functionalized textiles,^55^ where FTIR analysis mainly provides information on changes in the textile matrix rather than direct detection of low-concentration surface nanoparticles.

The FTIR results therefore suggest that SNP deposition on the cotton fabric occurs predominantly through non-covalent interactions rather than through the formation of new covalent bonds.^58^ These interactions may include physical adsorption and van der Waals forces between the TOAB-stabilized SNPs and the cellulose surface, together with hydrogen-bonding interactions involving cellulose hydroxyl groups and the bromide counterions associated with the TOAB stabilizer.^63^

### 3.4 Antifungal performance of treated and untreated fabrics

The antifungal performance of untreated and SNP-treated cotton fabrics was evaluated against *A. niger*, a filamentous fungus commonly associated with textile deterioration and frequently used as a test organism for evaluating antifungal activity of textile materials according to the AATCC TM 30 standard method.

**Fig. 11:**
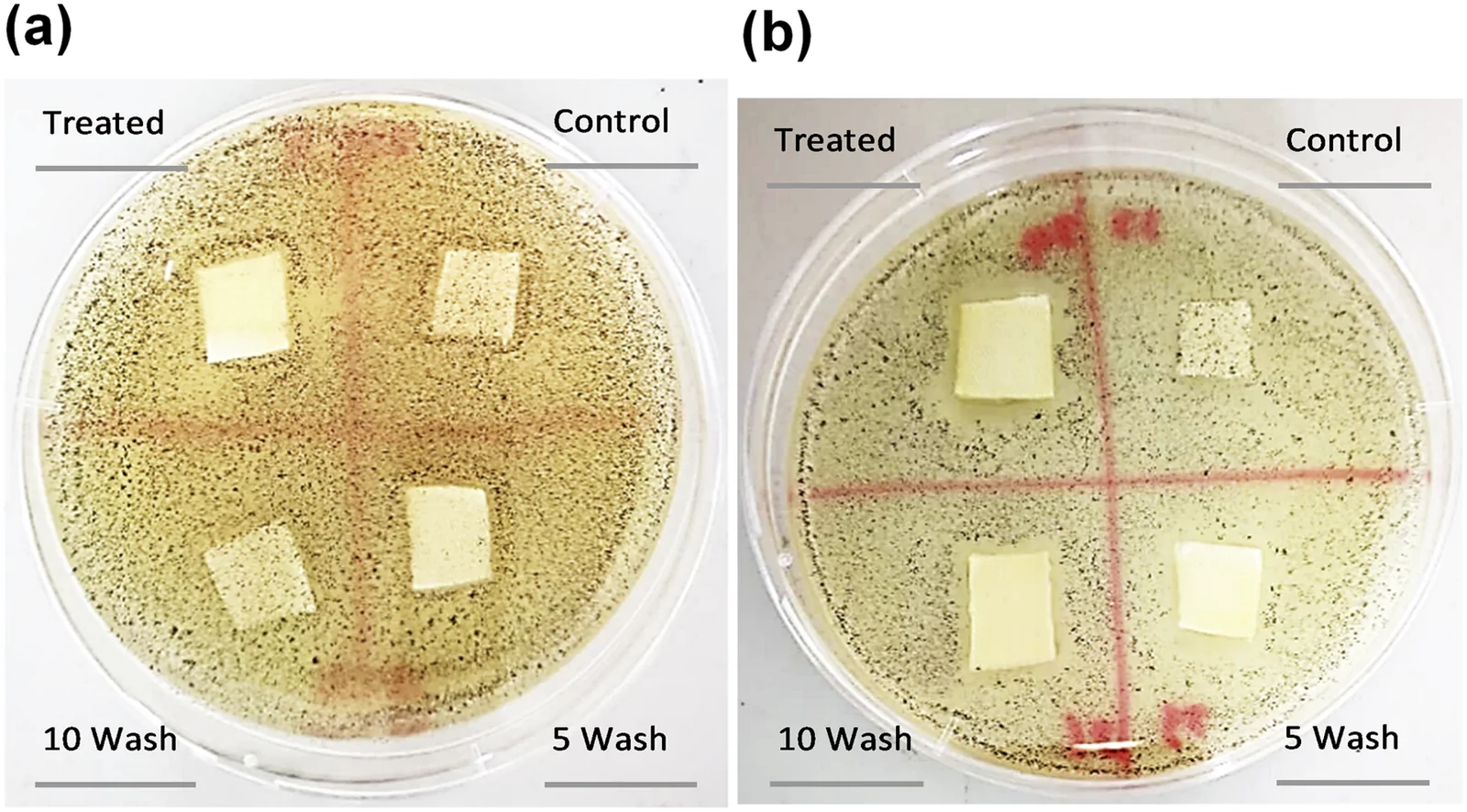
Antifungal activity of treated fabric studied by AATCC TM 30 method against *Aspergillus niger* (a) 1% SNPs and (b) 5% SNPs (owf).

Untreated cotton fabrics exhibited extensive fungal growth after incubation, confirming the susceptibility of cellulose-based materials to fungal colonization under favourable environmental conditions (Fig. 11). In contrast, SNP-treated fabrics showed clear inhibition of fungal growth, with no visible fungal colonization observed on the treated fabric surface. These results demonstrate that the incorporation of SNPs significantly improved the antifungal properties of cotton fabrics. The observed antifungal activity can be attributed to the intrinsic antifungal properties of SNPs. Previous studies have suggested that SNPs may inhibit fungal growth through multiple mechanisms, including interaction with the fungal cell surface, disruption of membrane integrity, alteration of cellular functions and induction of oxidative stress.^21^ However, the exact antifungal mechanism of SNPs remains dependent on factors such as nanoparticle size, surface chemistry and fungal species, and requires further mechanistic investigation. In the present study, the antifungal mechanism is therefore considered a proposed explanation rather than a directly confirmed pathway (Fig. S7, SI).

The retention of antifungal activity after immobilization onto cotton fibres indicates that the deposited SNP coating remained biologically active following textile functionalization. Importantly, this study demonstrates the successful application of TOAB-stabilized SNPs as a metal-free antifungal coating for cotton fabrics, addressing the limited exploration of SNPs in functional textile applications.

### 3.5 Effect of detergent on antifungal activity

In practical textile applications, laundering conditions can significantly influence the performance and durability of functional coatings. Therefore, evaluating the effect of washing conditions is essential to distinguish the intrinsic antifungal activity of the coating from any external factors associated with the washing process. To investigate the influence of detergent, control experiments were performed using untreated cotton fabrics subjected to washing in the presence of a commercial detergent. The washed untreated fabrics exhibited strong inhibition of *A. niger* growth regardless of the number of washing cycles, indicating that the detergent itself possessed antifungal activity (Fig. S8, SI). This observation demonstrates that the presence of detergent can interfere with the accurate evaluation of the antifungal performance of SNP-coated fabrics by contributing additional antimicrobial effects. Therefore, all subsequent washing durability experiments were conducted without detergent to ensure that the observed antifungal activity originated primarily from the SNP coating. This approach enabled a more reliable assessment of the intrinsic coating stability and antifungal retention after repeated laundering.

### 3.6 Antifungal performance of treated and untreated fabrics after washing

The effect of repeated washing cycles on the antifungal activity of SNP-treated cotton fabrics against *A. niger* is presented in Fig. 11.

Untreated cotton fabrics showed extensive fungal growth after incubation, confirming the susceptibility of untreated cellulose fibres to fungal colonization and demonstrating that the observed antifungal activity of treated fabrics was associated with the presence of SNPs.^64^ As shown in Fig. 11a, cotton fabrics treated with 1% SNPs (owf) retained antifungal activity up to five washing cycles; however, no visible inhibition was observed after further washing. In comparison, fabrics treated with 5% SNPs (owf) exhibited superior performance and maintained measurable antifungal activity even after 10 washing cycles (Fig. 11b). The improved durability at higher SNP loading can be attributed to the increased availability of nanoparticles on the fibre surface, which promotes greater nanoparticle retention and sustained antifungal activity during laundering.

**Fig. 12:**
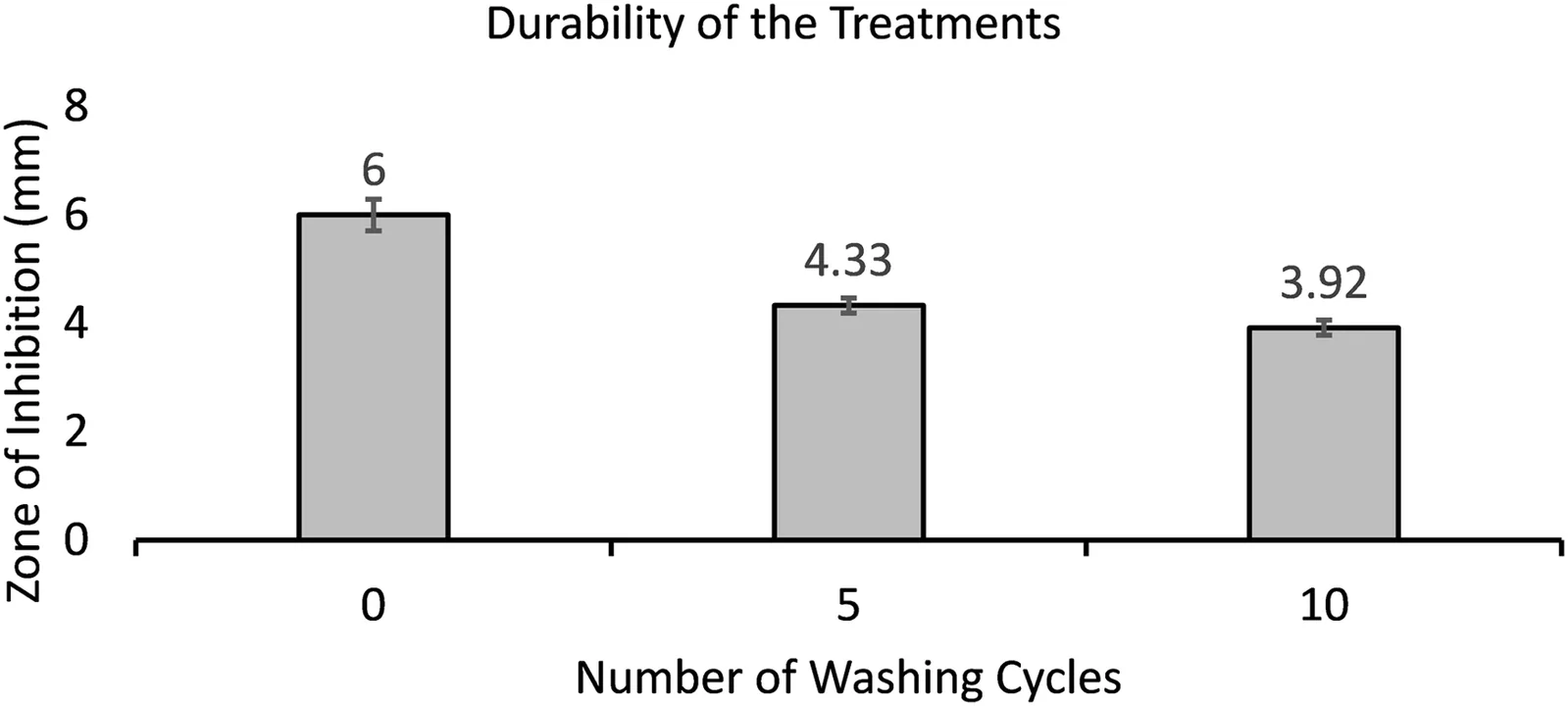
Antifungal activity (mean ± SD, *n* = 3) of cotton fabrics treated with 5% SNPs after 0, 5 and 10 washing cycles.

The quantitative comparison of antifungal performance based on inhibition zone diameter is summarized in Fig. 12.

The 5% SNP-treated fabrics showed a gradual reduction in inhibition zone from 6.00 ± 0.29 mm before washing to 3.92 ± 0.14 mm after 10 washing cycles. The reduction in activity with increasing washing cycles is likely associated with the removal of loosely attached nanoparticles and partial loss of surface coating during laundering. Nevertheless, the retention of measurable antifungal activity after repeated washing indicates that a fraction of SNPs remained associated with the cotton fibre surface and continued to provide antifungal protection. The relatively low standard deviation values demonstrate consistent performance among replicate samples.

These results align with previous studies that report the importance of binding strength and coating stability in maintaining antifungal activity of nanoparticles (AgNPs,^65,66^ ZnONPs,^23^ and TiO_2_ (ref. 67)) after washing.

Overall, the 5% SNP treatment provided the most effective balance between antifungal performance and washing stability among the tested formulations. These results demonstrate the potential of TOAB-stabilized SNPs as a promising metal-free antifungal finishing approach for cotton textiles.

### 3.7 Comparison of antifungal performance of selected nanoparticle-based cotton textile systems

The antifungal performance of the present SNP-treated cotton fabric was further contextualized by comparison with selected previously reported functionalized textile systems (Table 1).

**Table 1:** Comparison of antifungal performance of selected nanoparticle-based and functionalized cotton textile systems against *A. niger*

Antifungal treatment	Inhibition zone (mm) against *A. niger*	Reference
TOAB-stabilized SNPs	6.0	Present study
AgNPs coating	30.2	68
ZnO NP coating	14.0	69
TiO_2_–SiO_2_/chitosan coating	12.9	67
Aloe vera/silane coating	13.0	70
CuO NP coating	20.5	71

The inhibition zone obtained for the present TOAB-stabilized SNP treatment was lower than those reported for some metal-based nanoparticle systems, such as AgNP and ZnONP-based cotton fabrics. However, direct comparison of inhibition-zone diameters should be interpreted with caution because the reported values are strongly influenced by nanoparticle concentration, loading level, textile substrate, coating method, fungal inoculum, incubation conditions and the specific antifungal testing protocol. Therefore, the numerical values should not be considered as a direct ranking of antifungal efficacy among different textile systems.

The present study nevertheless demonstrates that a metal-free sulfur-based coating can provide measurable antifungal activity against *A. niger* while retaining activity after repeated laundering. In particular, the 5% owf SNP-treated fabric retained an inhibition zone of 3.92 ± 0.14 mm after 10 washing cycles, compared with 6.00 ± 0.29 mm before washing. This persistence of activity is important because the objective of the present work was not to achieve the highest instantaneous inhibition zone, but to investigate the feasibility of a sulfur-based, metal-free antifungal finish for cotton textiles with washing durability. Compared with metal-containing systems, the sulfur-based approach may offer an alternative route for developing functional textiles where avoidance of metal-based antimicrobial agents is desirable. Nonetheless, the relatively smaller inhibition zones obtained in the present study indicate that further optimization of SNP loading, particle–fibre interactions and coating adhesion is required to enhance antifungal efficacy and long-term durability.

## 4 Conclusions

The SNP-based coating developed in this study demonstrated strong antifungal performance on cotton fabrics, with significant inhibition of fungal growth and spore formation. The effectiveness of the coating was influenced by the method of SNP preparation and application, which affected nanoparticle distribution, adhesion, and overall durability. In particular, the treated fabrics retained substantial antifungal activity after repeated washing cycles, indicating that the coating achieved a reasonable level of binding to the fibre surface. Compared to conventional antifungal treatments, the SNP approach provides a promising alternative due to its low cost, reduced toxicity, and environmental compatibility. The nanoscale formulation enhances the reactivity of sulfur, enabling effective antifungal action at relatively low concentrations, while avoiding the limitations associated with metal-based systems such as silver nanoparticles. Given the promising antifungal performance and durability observed, this work highlights the potential of SNP-coated cotton fabrics for applications requiring hygienic and long-lasting textile materials. Further studies should focus on optimizing nanoparticle–fibre interactions to improve wash durability, including the use of advanced binders or covalent attachment strategies. It would also be beneficial to investigate the establishment of broader antifungal activity and real-world performance under practical usage conditions to fully strengthen the applicability of this approach.

## Author contributions

Nirasha Jayawardhana: formal analysis, data curation, visualization, writing – original draft preparation, Kasindu Pramod: methodology, validation, resources, Characterization, writing – review & editing, Danushika C. Manatunga: supervision, validation, writing – review & editing and Nuwan De Silva: conceptualization, supervision, resources, project administration, validation, writing – review & editing.

## Conflicts of interest

The authors declare no conflicts of interest.

## Supplementary Material

RA-OLF-D6RA05516H-s001

## Data Availability

The data supporting this article have been included as part of the supplementary information (SI). Supplementary information is available. See DOI: https://doi.org/10.1039/d6ra05516h.

## References

[cit1] Khanzada H. , Khan M. Q. and Kayani S., in Cotton Science and Processing Technology: Gene, Ginning, Garment and Green Recycling, ed. H. Wang and H. Memon, Springer Singapore, Singapore, 2020, pp. 377–391, 10.1007/978-981-15-9169-3_15

[cit2] Hosseini Ravandi S. A. and Valizadeh M., in Improving Comfort in Clothing, Elsevier, 2011, pp. 61–78, 10.1533/9780857090645.1.61

[cit3] Surati B. I., Minocheherhomji D. F. P. (2025). J. Biol. Sci..

[cit4] Al-Shaarani A. A. Q. A., Pecoraro L. (2024). Front. Microbiol..

[cit5] Wang C., Wei X., Zhong L., Chan C. L., Li H., Sun H. (2025). J. Am. Chem. Soc..

[cit6] Islam S. U., Sun G. (2022). ACS Sustainable Chem. Eng..

[cit7] Mustafa S. K., Omer N., Aljohani M. M. H., Alessa A. H., Jame R., Alatawi A. O., Omran A. M. E., Alatawi O. M., Sagheer M., Islam M., Umar K., Pandey S. (2024). Inorg. Chem. Commun..

[cit8] Xu L., Wang Y. Y., Huang J., Chen C. Y., Wang Z. X., Xie H. (2020). Theranostics.

[cit9] Das D., Paul P. (2025). Plant Nano Biol..

[cit10] Dąbrowska-Bouta B., Sulkowski G., Strużyński W., Strużyńska L. (2019). Neurotoxic. Res..

[cit11] Akter M., Sikder M. T., Rahman M. M., Ullah A. K. M. A., Hossain K. F. B., Banik S., Hosokawa T., Saito T., Kurasaki M. (2017). J. Adv. Res..

[cit12] Morozova O. V. and Klinov D. V., in Silver Micro-nanoparticles – Properties, Synthesis, Characterization and Applications, ed. S. K. Kumar, P. Kumar and C. S. Pathak, IntechOpen, 2021, ch. 6, 10.5772/intechopen.96331

[cit13] Uddin F. (2014). Int. J. Text. Sci..

[cit14] Saedi S., Shokri M., Rhim J. W. (2020). Arab. J. Chem..

[cit15] Chen Y. J., Tang J., Wang L., Hua W. (2025). J. Cosmet. Dermatol..

[cit16] Wijeyatunga S. K., Sauceda-Oloño P. Y., Kapuge Dona N. L., Guinati B. G. S., Derr K. M., Tisdale K. A., Smith A. D., Tennyson A. G., Smith R. C. (2025). Molecules.

[cit17] Priyadarshi R., Kim H. J., Rhim J. W. (2021). Food Hydrocolloids.

[cit18] Roy Choudhury S., Ghosh M., Mandal A., Chakravorty D., Pal M., Pradhan S., Goswami A. (2011). Appl. Microbiol. Biotechnol..

[cit19] Rai M., Ingle A. P., Paralikar P. (2016). Expert Rev. Anti-Infect. Ther..

[cit20] Sadek M. E., Shabana Y. M., Sayed-Ahmed K., Abou Tabl A. H. (2022). J. Fungi.

[cit21] Choudhury S. R. and Goswami A., arXiv, 2015, preprint, arXiv:1501.02409, 10.48550/arXiv.1501.02409

[cit22] Jalali E., Erasmus E., Schutte-Smith M., Visser H. G. (2024). Mater. Today Commun..

[cit23] Tania I. S., Ali M. (2021). Polymers.

[cit24] Zhou J., Cai D., Xu Q., Zhang Y., Fu F., Diao H., Liu X. (2018). RSC Adv..

[cit25] Gulati R., Sharma S., Sharma R. K. (2022). Polym. Bull..

[cit26] Tania I. S., Ali M., Akter M. (2022). J. Eng. Fibers Fabr..

[cit27] Rajput S. K., Singh M. K., Shakyawar D. B. (2023). Fibres Text. East. Eur..

[cit28] Xiao Q., Xie E., Guo L., Wang W. (2025). Int. J. Cloth. Sci. Technol..

[cit29] Nguyen-Tri P., Altiparmak F., Nguyen N., Tuduri L., Ouellet-Plamondon C. M., Prud’homme R. E. (2019). ACS Omega.

[cit30] Shilpa S. A., Arthi C., Sobhin Hikku G. S., Jeyasubramanian K., Veluswamy P., Ikeda H. (2024). Biointerface Res. Appl. Chem..

[cit31] Suleiman M., Al-Masri M., Al Ali A., Aref D., Hussein A., Saadeddin I., Warad I. (2015). J. Mater. Environ. Sci..

[cit32] Asmat-Campos D., Delfín-Narciso D., Juárez-Cortijo L. (2021). Fibers.

[cit33] Elmaaty T. A., Sayed-Ahmed K., Ali R. M., El-Khodary K., Abdeldayem S. A. (2022). Polymers.

[cit34] American Association of Textile Chemists and Colorists (AATCC) , AATCC Manual of International Test Methods and Procedures, AATCC, 100th edn, 2025

[cit35] Simončič B., Rozman V. (2007). Colloids Surf., A.

[cit36] Xie X.-Y., Li L.-Y., Zheng P.-S., Zheng W.-J., Bai Y., Cheng T.-F., Liu J. (2012). Mater. Res. Bull..

[cit37] Sahoo R., Mundamajhi A., Das S. K. (2019). J. Mater. Sci.: Mater. Electron..

[cit38] Dixit N. S., Khobragade S., Dixit M. S., Dixit A., Ardak P. (2025). Int. J. Sci. Res. Sci. Technol..

[cit39] Liu X., Zhu K., Tian J., Tang Q., Shan Z. (2014). J. Solid State Electrochem..

[cit40] Turganbay S., Aidarova S. B., Bekturganova N. E., Alimbekova G. K., Musabekov K. B., Kumargalieva S. S. (2013). Adv. Mater. Res..

[cit41] Chaudhuri R. G., Paria S. (2010). J. Colloid Interface Sci..

[cit42] Turganbay S., Aidarova S. B., Turganbay G., Tileuberdi Y., Chen S. L. (2019). Int. J. Biol. Chem..

[cit43] Bokuniaeva A. O., Vorokh A. S. (2019). J. Phys.: Conf. Ser..

[cit44] Maqsoodi M., Tajbakhsh E., Momtaz H. (2025). BMC Biotechnol..

[cit45] Khlebtsov B. N., Khlebtsov N. G. (2011). Colloid J..

[cit46] Dai Y. X., Li Y. X., Zhang X. J., Cosnier S., Shan D. (2023). ACS Appl. Mater. Interfaces.

[cit47] Jeon Y. N., Ryu S. J., Sathiyaseelan A., Baek J. S. (2025). Bioinorg. Chem. Appl..

[cit48] Xu R. (2008). Particuology.

[cit49] Mourdikoudis S., Pallares R. M., Thanh N. T. K. (2018). Nanoscale.

[cit50] Mahltig B., Grethe T. (2022). Textiles.

[cit51] Mandal S., Arumugam S. K., Adyanthaya S. D., Pasricha R., Sastry M. (2004). J. Mater. Chem..

[cit52] Celik S., Albayrak A. T., Akyuz S., Ozel A. E. (2019). J. Biomol. Struct. Dyn..

[cit53] Shestakov A. F., Konarev D. V., Simonov S. V., Khasanov S. S., Lapshin A. N., Goldshleger N. F. (2013). RSC Adv..

[cit54] Alwan B. J., Nsaif N. H., Abd Al-Sada Z. F., Al-Baraji G. A. M., Haddawi S. F. (2025). J. Theor. Appl. Phys..

[cit55] Arık B., Seventekin N. (2011). Tekst. ve Konfeksiyon..

[cit56] Abbaszadegan A., Ghahramani Y., Gholami A., Hemmateenejad B., Dorostkar S., Nabavizadeh M., Sharghi H. (2015). J. Nanomater..

[cit57] Cho E. C., Xie J., Wurm P. A., Xia Y. (2009). Nano Lett..

[cit58] Wei Y., Wu J., Ren Z., Sun P., Zhang S., Xu M. (2026). J. Mater. Res. Technol..

[cit59] Mehrizi M. K., Mortazavi S. M., Mallakpour S., Bidoki S. M. (2012). Color. Res. Appl..

[cit60] Arık B., Karaman Atmaca O. D. (2020). Cellulose.

[cit61] Tong J., Vo Q. N. N., He X., Liu H., Zhou H., Park C. H. (2024). Int. J. Biol. Macromol..

[cit62] Cichosz S., Masek A. (2020). Materials.

[cit63] Zhong C., Cheng F., Zhu Y., Gao Z., Jia H., Wei P. (2017). Carbohydr. Polym..

[cit64] Arenas-Chávez C. A., de Hollanda L. M., Arce-Esquivel A. A., Alvarez-Risco A., Del-Aguila-Arcentales S., Yáñez J. A., Vera-Gonzales C. (2022). Processes.

[cit65] Xu Q. B., Xie L. J., Diao H., Li F., Zhang Y. Y., Fu F. Y., Liu X. D. (2017). Carbohydr. Polym..

[cit66] Jain A., Kongkham B., Puttaswamy H., Butola B. S., Malik H. K., Malik A. (2022). Antibiotics.

[cit67] Rilda Y., Dwiyanti D., Syukri S., Agustien A., Pardi H. (2021). J. Dispersion Sci. Technol..

[cit68] Elbasuney S., El-Sayyad G. S. (2022). Microb. Pathog..

[cit69] Roy T. S., Shamim S. U. D., Rahman M. K., Ahmed F., Gafur M. A. (2020). Mater. Sci. Appl..

[cit70] Chauhan P., Kumar A. (2020). Carbohydr. Polym. Technol. Appl..

[cit71] Moges G., Haile A., Pawlos M. (2025). Results Surf. Interfaces.

