# Supplementary material for: Development of durable sulfur nanoparticle-based antifungal coatings for cotton fabrics
Source: RSC Adv. 2026 Sep 21;16(44):55939–51. doi: 10.1039/d6ra05516h (PMC13591316; doi:10.1039/d6ra05516h)
Supplement: Supplementary file 1 [file RA-016-D6RA05516H-s001.pdf]

Supplementary Information (SI) for RSC Advances.
This journal is © The Royal Society of Chemistry

Development of Durable Sulfur Nanoparticle-Based Antifungal Coatings for Cotton Fabrics

Nirasha Jayawardhana,[a] Kasindu Pramod,[b] Danushika Manatunga[*a] and Nuwan De Silva[b]

[a]Department of Biosystems Technology, Faculty of Technology, University of Sri Jayewardenepura, Homagama 10206, Sri Lanka. E-mail: danushi@sjp.ac.lk.

[b]Sri Lanka Institute of Nanotechnology (Pvt) Ltd (SLINTEC), Homagama 10206, Sri Lanka.

Supplementary Information

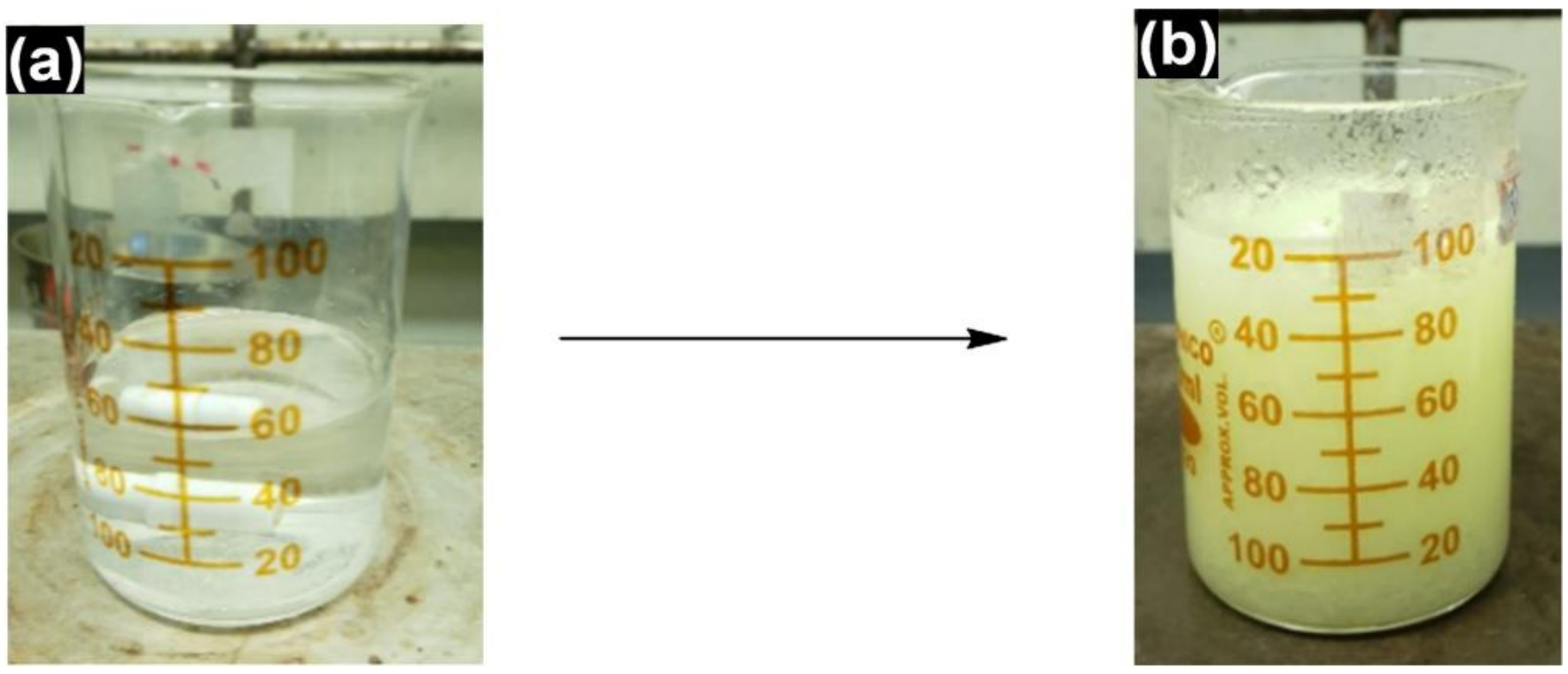

Fig. S1 Colour change during SNPs formation (a) initial colour and (b) colour after reaction.

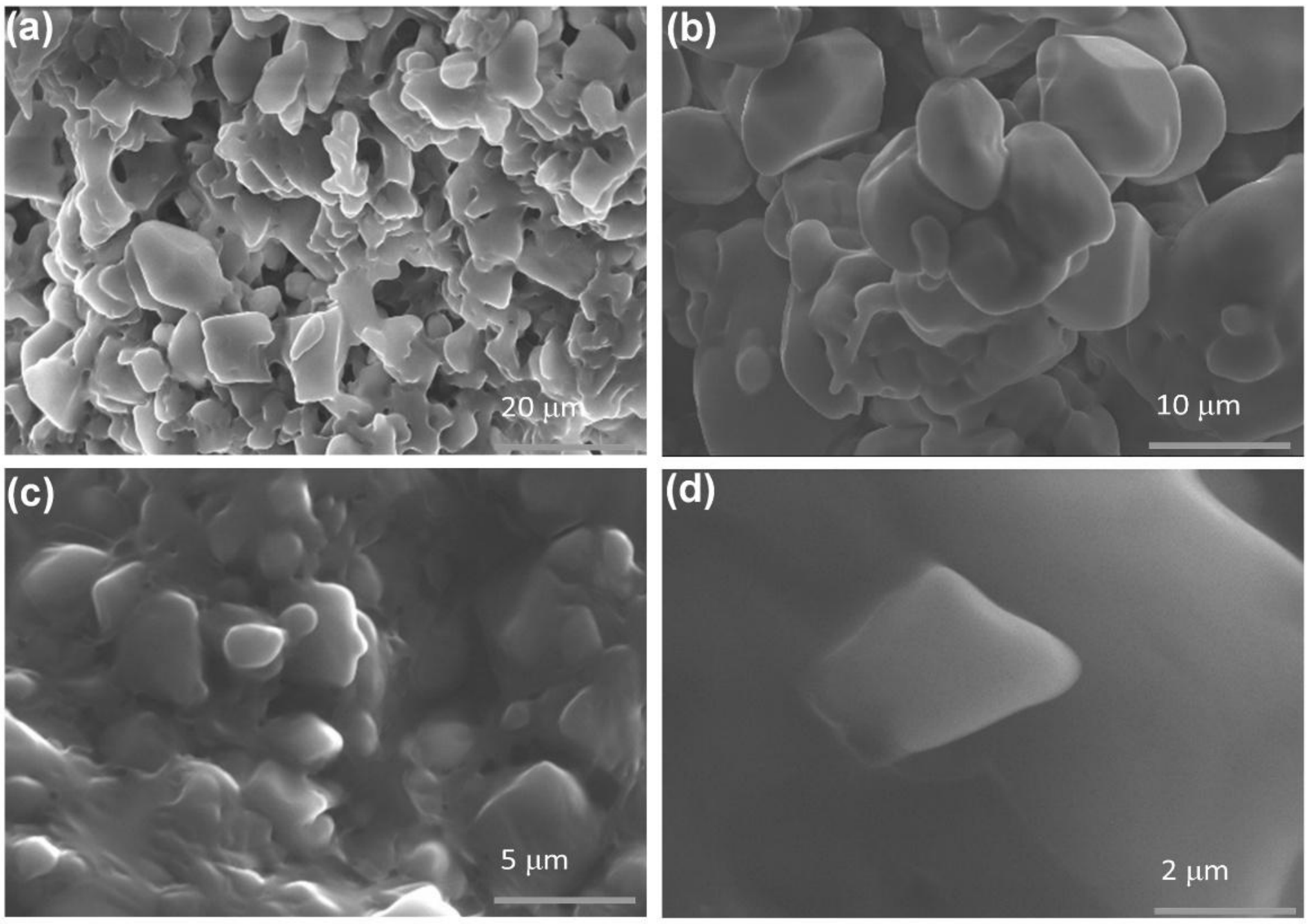

Fig. S2 SEM images of SNPs under magnifications of (a) 20 µm, (b) 10 µm, (c) 5 µm and (d) 2 µm.

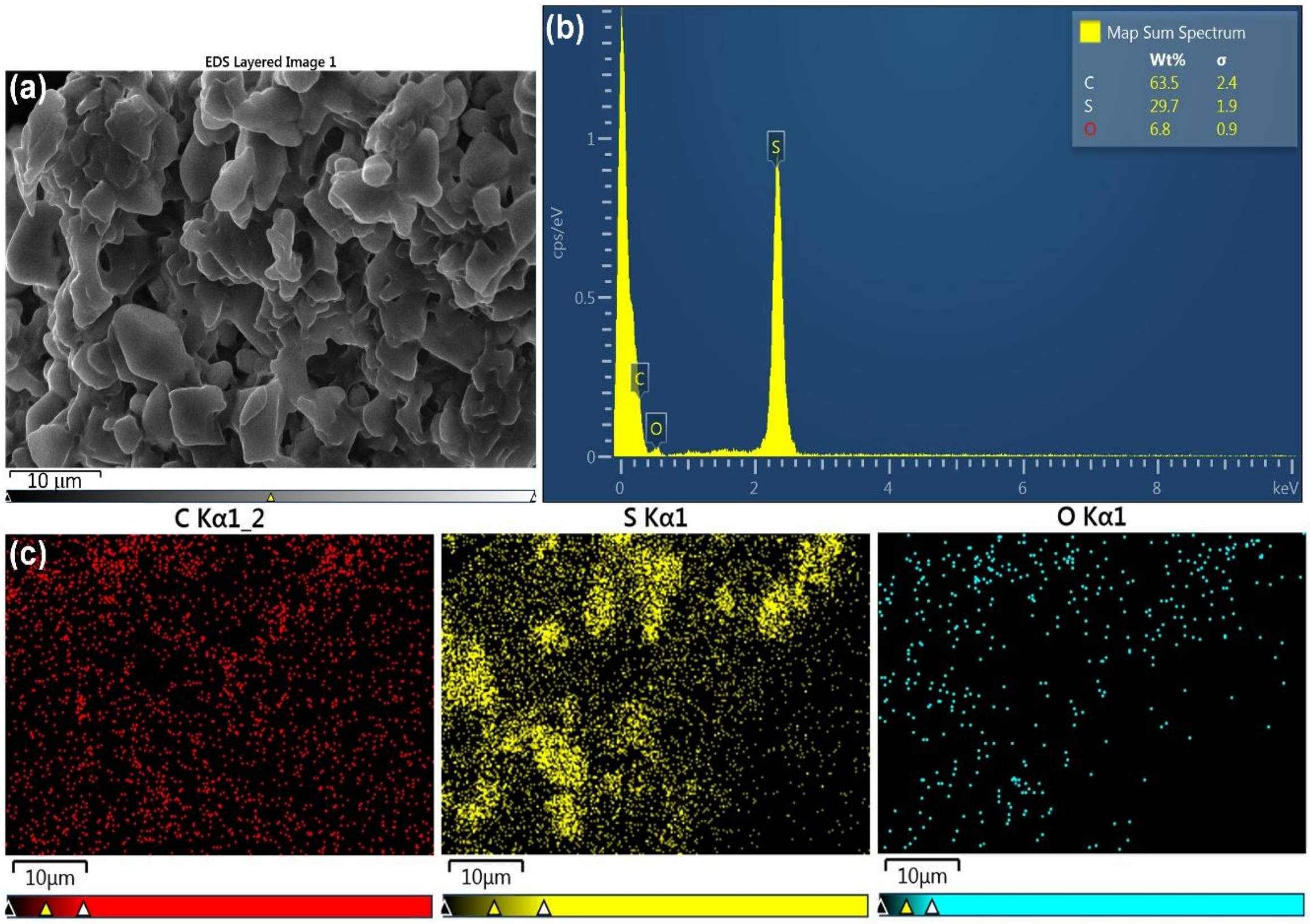

Fig. S3 SEM-EDS analysis and elemental mapping of the SNPs (a) SEM image, (b) EDS spectra and (c) elemental maps showing carbon (red), sulfur (yellow) and oxygen (blue).

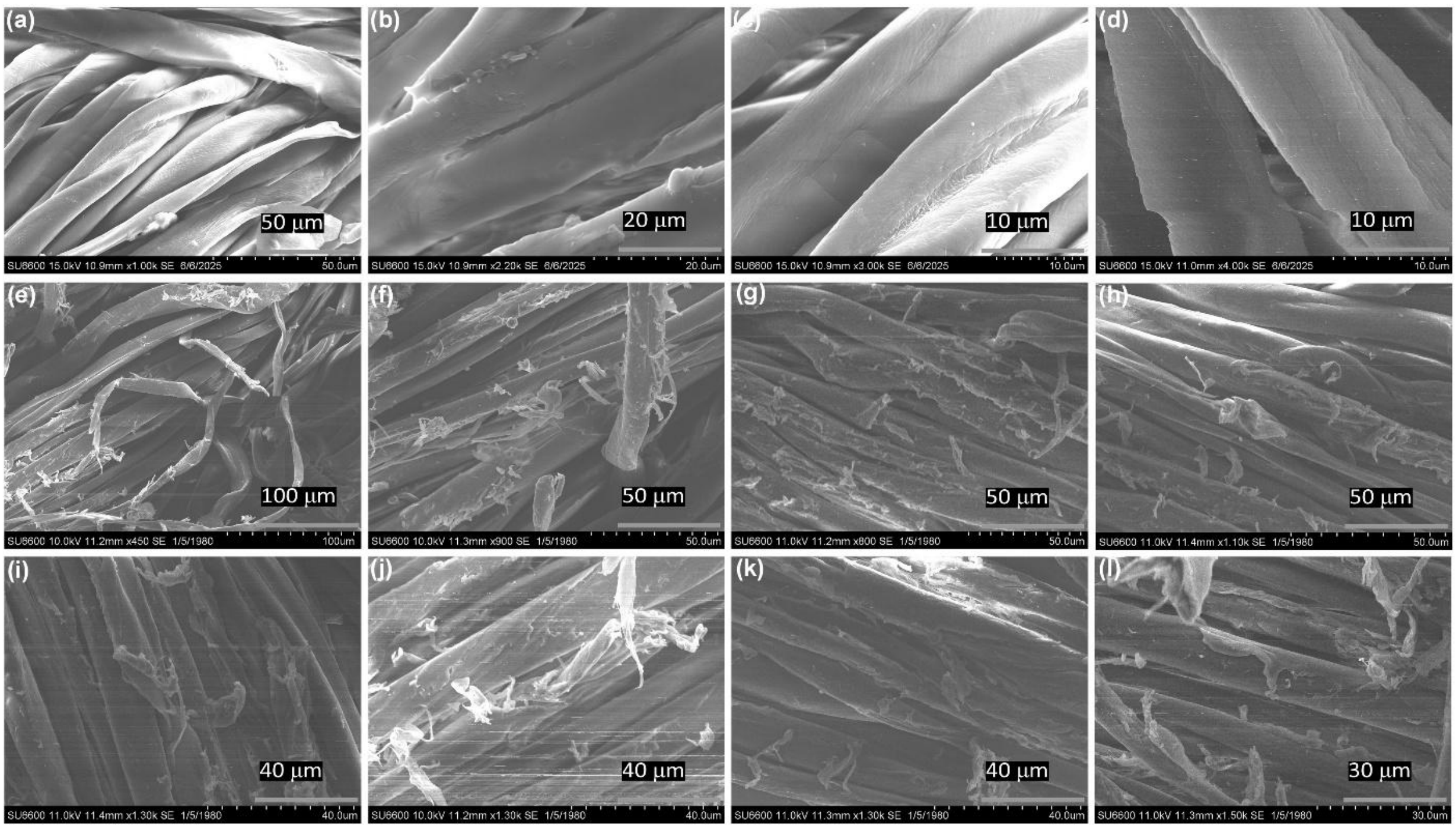

Fig. S4 SEM images of fabrics at different magnifications (a-d) treated fabrics with SNPs and (e-l) treated Fabrics after10 washing cycles.

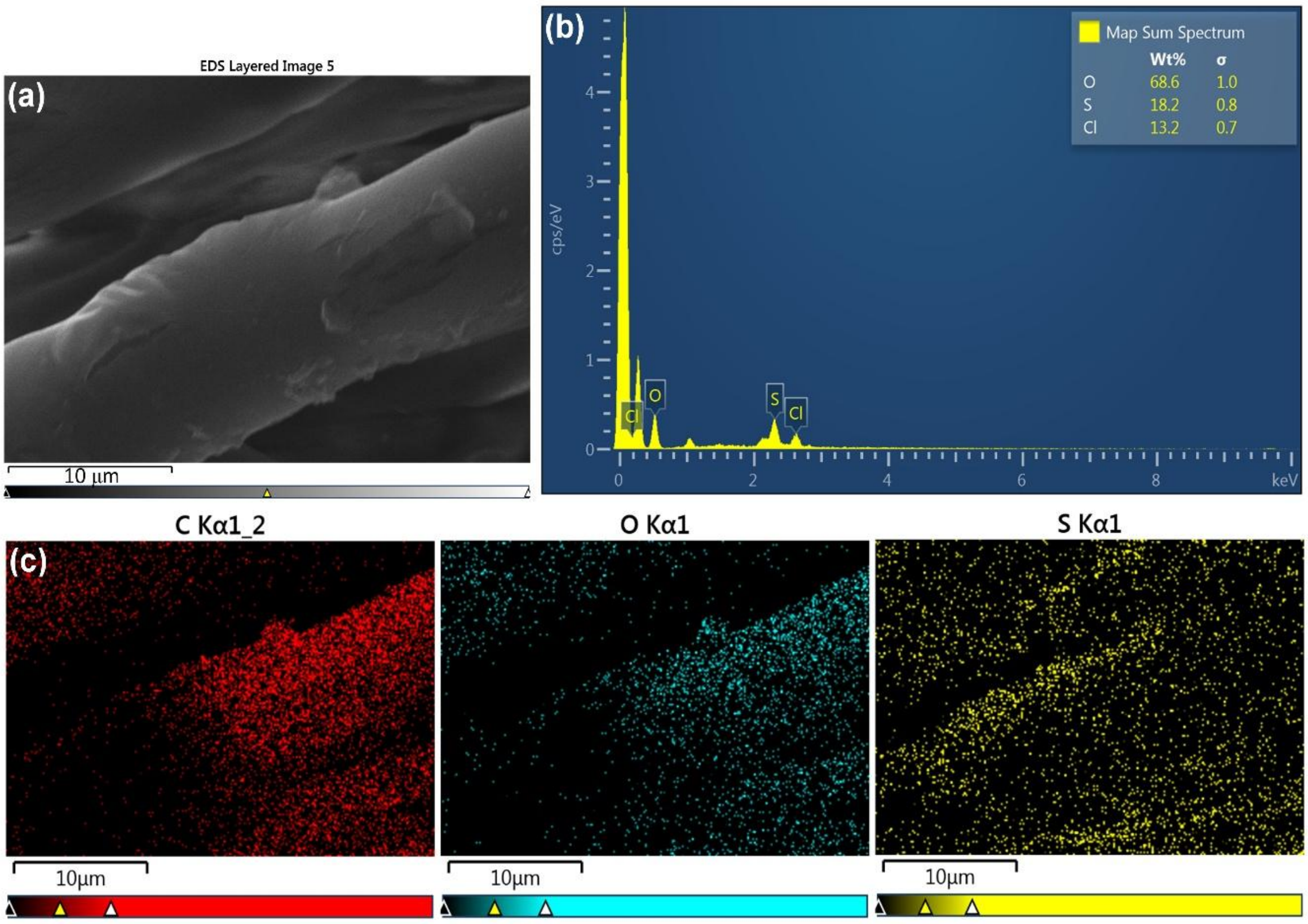

Fig. S5 SEM-EDS analysis of SNP-treated cotton fabric (a) SEM image, (b) EDS spectrum and (c) elemental maps of the SNP-treated fabric, showing the distribution of carbon (red), oxygen (blue) and sulfur (yellow).

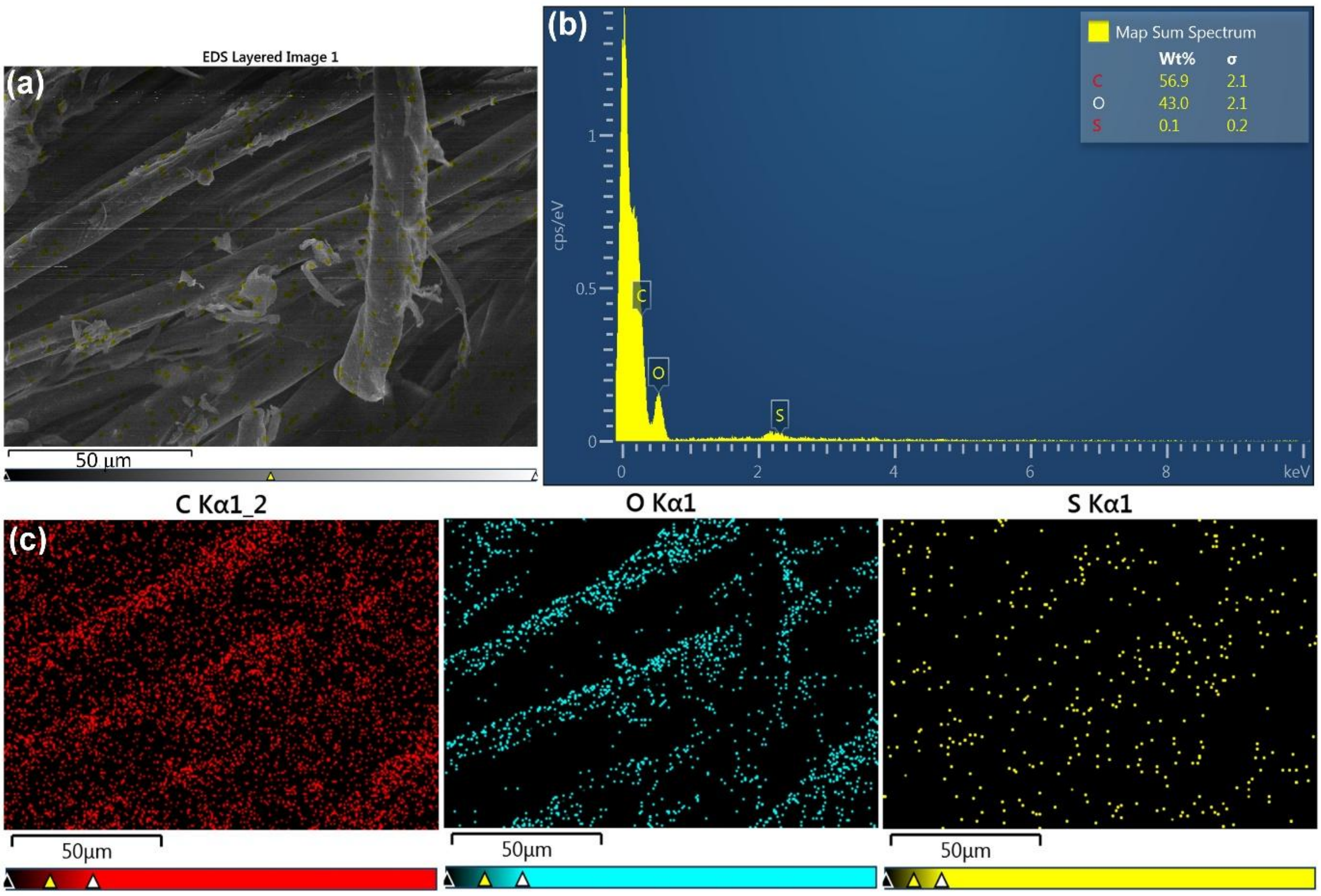

Fig. S6 SEM-EDS analysis and elemental mapping of 10 times washed SNP treated cotton fabric (a) SEM image, (b) EDS spectra and (c) elemental maps showing carbon (red), oxygen (blue) and sulfur (yellow).

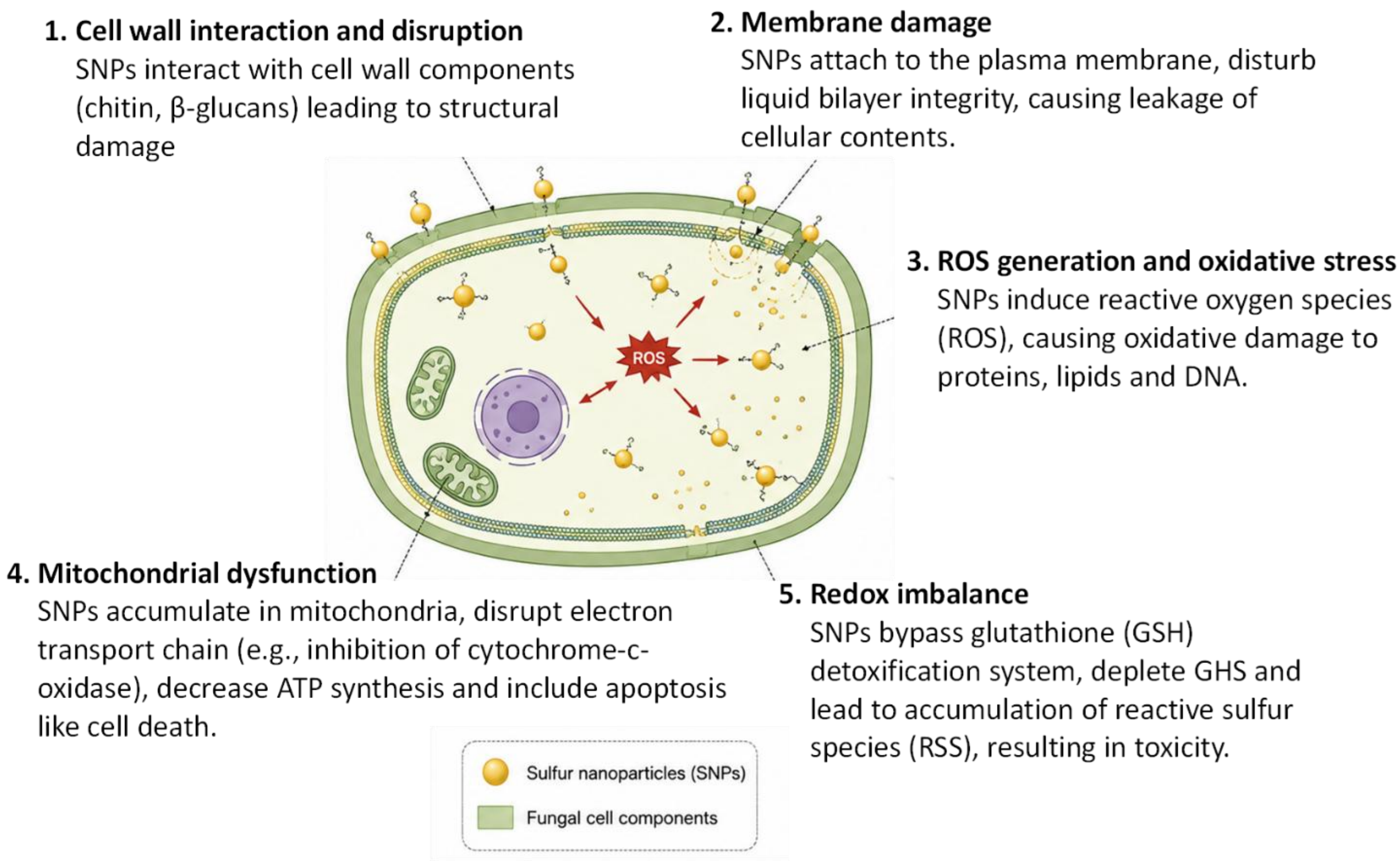

Fig. S7 Proposed antifungal mechanism of SNP coating.

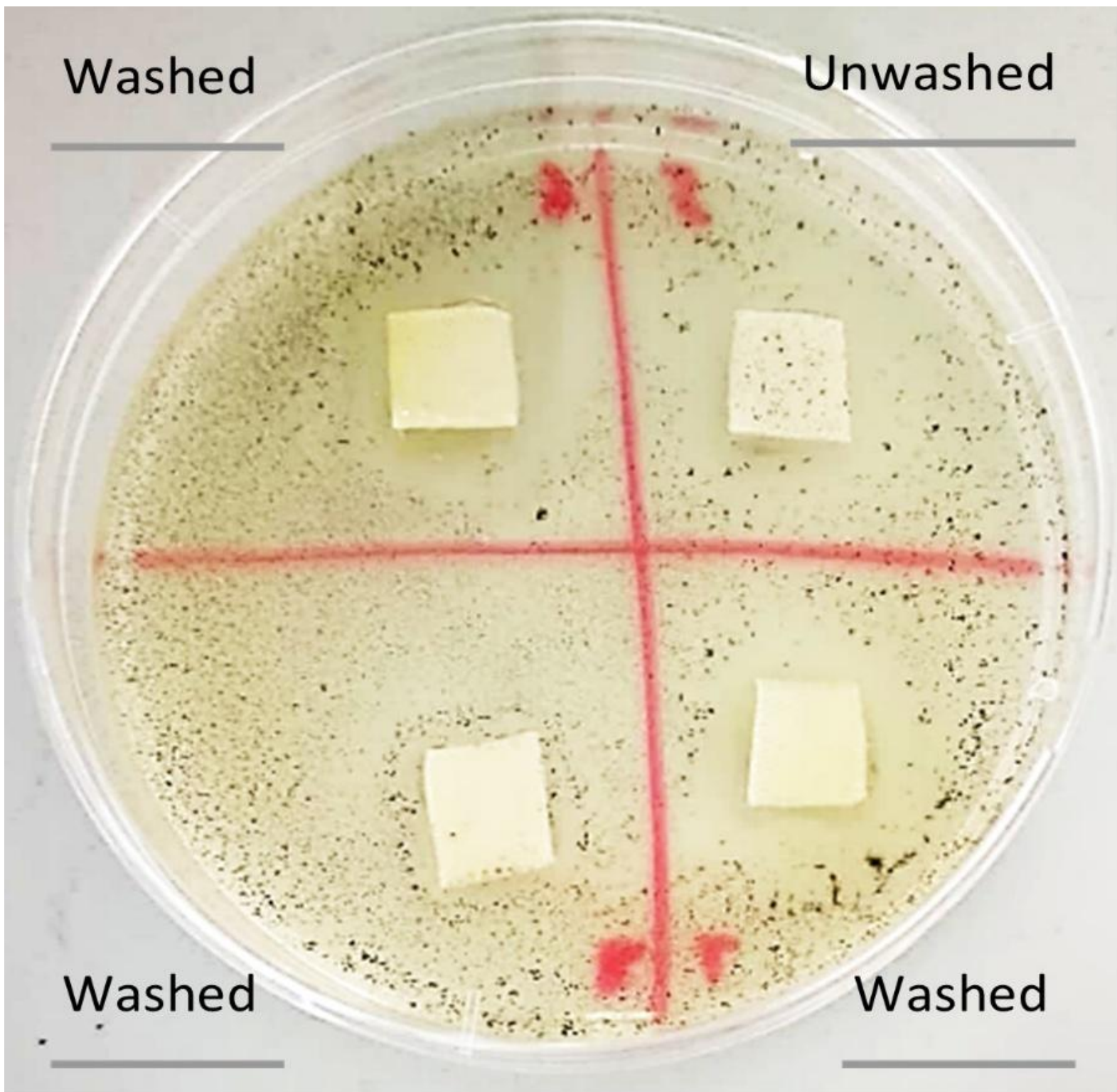

Fig. S8 Antifungal activity of untreated cotton fabrics washed with commercial detergent against *A. niger* after different washing cycles.
